# The Potential of Microwave Radiation and Mechanochemistry in the Formation of Purine Alkaloids Cocrystals Using Pyromellitic Acid as a Coformer: Synthesis, Structural, Spectroscopic, Thermal Analysis, and Biological Properties

**DOI:** 10.1021/acs.cgd.5c00860

**Published:** 2025-08-27

**Authors:** Weronika Cal, Mateusz R. Gołdyn, Oliwia Grupa, Justyna Starzyk, Daria Larowska-Zarych, Kamil Frąckowiak, Elżbieta Bartoszak-Adamska

**Affiliations:** † Faculty of Chemistry, 49562 Adam Mickiewicz University in Poznań, Uniwersytetu Poznańskiego 8, Poznań 61-614, Poland; ‡ Center for Advanced Technologies, Adam Mickiewicz University in Poznań, Uniwersytetu Poznańskiego 10, Poznań 61-614, Poland; § Faculty of Agronomy, Horticulture, and Bioengineering, University of Life Science, Szydłowska 50, Poznań 60-656, Poland; ∥ Institute of Physical Chemistry, 49559 Polish Academy of Sciences, Kasprzaka 44/52, Warsaw 01-224, Poland

## Abstract

Cocrystallization provides an efficient approach to modifying a wide range of physicochemical properties of active pharmaceutical ingredients (APIs), including solubility, dissolution rate, melting point, and hygroscopicity. Therefore, the development of effective and fast cocrystallization techniques is crucial for selectively obtaining a specific crystalline form. This study explores the potential of green chemical methods for synthesizing multicomponent cocrystals of theobromine (TBR) and theophylline (TPH) using pyromellitic acid (PMLA) as a coformer. Solution-based screening experiments with TBR resulted in the identification of a new TBR·PMLA 2:1 cocrystal. In the case of TPH, four new multicomponent forms were discovered, including two polymorphic TPH·PMLA 2:1 cocrystals (forms I and II) and two cocrystal solvates: TPH·PMLA·MeOH 2:1:2 and TPH·PMLA·H_2_O 1:1:2. Cocrystallization via grinding enabled the formation of cocrystals within 30 min to 2 h, while microwave-assisted cocrystallization significantly reduced the process time to just 5 min. Powder X-ray diffraction (PXRD) confirmed the formation of the obtained cocrystals, and single-crystal X-ray diffraction (SXRD) facilitated X-ray structural analysis for the characteristic supramolecular synthons formation in the crystal. Simultaneous thermal analysis (STA) demonstrated the high thermal stability of the studied systems. Additionally, a variable-temperature SXRD experiment, performed for the TPH·PMLA·MeOH 2:1:2 single crystal in the 300–415 K range, revealed negative volumetric thermal expansion of this cocrystal solvate and a gradual solvent release, ultimately leading to a phase transition into the TPH·PMLA 2:1 II cocrystal. UV–vis spectroscopy confirmed an enhancement in TBR solubility and a decrease in TPH solubility in water following cocrystallization using PMLA. Furthermore, biological studies demonstrated the influence of the cocrystallization on the inhibition of specific bacterial and fungal strains.

## Graphical abstract

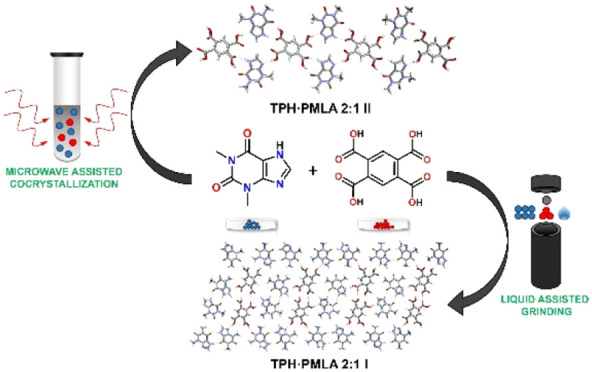

## 1 Introduction

In recent years, there has been a significant increase in interest in the pharmaceutical engineering of cocrystals. Cocrystals are multicomponent molecular crystals in which all components are in a stoichiometric ratio and, additionally, are solids in ambient conditions.^1^ None of the components can act as a solvent in the cocrystallization process.^2^ Cocrystal design involves the formation of supramolecular hetero- or homosynthons, composed of specific functional groups, such as carboxyl, amino, amide, or hydroxyl groups, which are connected by noncovalent bonds.^3^ Supramolecular heterosynthons–intermolecular interactions between different complementary functional groups are statistically more prevalent and energetically favored over homosynthons, which involve identical functional groups pairing.^4^ This preference arises because heterosynthons typically form stronger, more directional hydrogen bonds, which enhance lattice energy optimization and thus promote cocrystal formation.^5^ These interactions more reliably lead to thermodynamically stable cocrystals where optimal hydrogen bond networks and efficient molecular packing drive crystallization.

The basic cocrystallization technique, cocrystallization by slow evaporation, is a method that involves dissolving the coformers in a suitable liquid medium, followed by evaporation of the solvent. As evaporation progresses, supersaturation is generated, leading to cocrystal nucleation and growth. It is a widely used experimental screening method due to its simplicity and efficiency in determining the appropriate conditions for cocrystal formation.^6^ The solvent evaporation method is suitable for cocrystallization in a small volume of a solvent or a mixture of solvents and can be easily set up and monitored. However, this method can also lead to the precipitation or crystallization of pure components or eutectic solid mixtures and the undesirable formation of solvates. They can be identified by routine analytical techniques such as X-ray crystallography or differential scanning calorimetry.^7^

In the grinding process, cocrystals are formed through a mechanochemical reaction induced solely by mechanical energy.^8^ This process entails loading the raw materials into a rotating chamber partially filled with a grinding medium, typically ceramic or stainless steel balls, and cocrystallization occurs by reducing the particle size through impact.^9^ It has been found that cocrystals that cannot be obtained by dissolution methods can be produced by mechanochemical synthesis. The main advantage of neat grinding, a solvent-free method, is screening API cocrystals composed of coformers with low solubility or those with a significant difference in solubility. However, during the dry milling process, there is a possibility of incomplete conversion of coformers due to insufficient energy needed to complete the cocrystallization process.^10^ In contrast to neat grinding, in liquid-assisted grinding, a small volume of liquid medium is introduced into the milling jar, which acts as a catalyst to support the entire process.^11^

Microwave radiation is a valuable tool for cocrystal synthesis, as it facilitates the formation of smaller particles in a shorter time compared with traditional thermal methods. In microwave-assisted slurry crystallization, microwave heating enhances the molecular motion, increasing the likelihood of molecular collisions and interactions between solute and solvent molecules. Specifically, microwave radiation promotes the ordering and alignment of molecules following the frequency of the electromagnetic field, which is likely a key factor in the accelerated cocrystal formation process.^12^ Studies have shown that when crystallizing the conversion slurry at the same elevated temperature, microwaves as a heat source significantly increase the rate of cocrystal formation in comparison to conventional heating. It appears that the alternating electromagnetic field contributes to the solid-phase transition beyond the effect of temperature increase alone.^7^

The topic of cocrystallization of purine alkaloids using various coformers remains relevant, as shown by numerous scientific papers.^13–22^ The potential to enhance physicochemical properties, such as solubility and stability, is appealing from a pharmaceutical perspective and helps make these substances more attractive for broader use. This paper presents the use of grinding and microwave radiation to obtain a series of multicomponent purine alkaloid systems using pyromellitic acid as a coformer. Pyromellitic acid is a safe and effective coformer for cocrystallization, offering excellent hydrogen bonding capability and the ability to form varied cocrystals, salts, solvates, and clathrates.^23–26^ The tendency to form diverse structures is due to its four carboxylic acid groups, which enable the formation of interesting supramolecular motifs rich in diverse hydrogen bonds, which is extremely interesting from the point of view of crystal engineering and cocrystal design. Its use is known in the synthesis of pharmaceutical cocrystals with different APIs and biologically active materials, like MOFs.^27–31^ The conditions for selectively obtaining five multicomponent systems by using these techniques were developed. This required selecting the appropriate stoichiometric ratio of substrates, volume and type of liquid medium, and reaction time. X-ray experiments confirmed the obtaining of pure phases and allowed for the structural analysis of novel multicomponent systems.

So far, few purine alkaloid systems have been published using this technique. These include theophylline-acetylsalicylic acid,^32^ caffeine-maleic acid,^33^ and caffeine-caffeic acid phenylethyl ester.^34^ In one of our earlier works, we also described the use of microwave-assisted slurry cocrystallization to obtain theobromine and caffeine salts of 2,6-dihydroxybenzoic acid.^35^ Concerning caffeine-maleic acid cocrystals in the ratios of 1:1 and 2:1, several papers describe different selective methods for their preparation.^17,33,36–39^ The above indicates the necessity and validity of continuing research on microwave-assisted cocrystallization, not only about purine alkaloids but also on many other pharmaceutical substances, which will undoubtedly contribute to a better understanding of the processes of self-assembly of molecules under the influence of microwave radiation. Moreover, this method, as shown by our studies, allows for simultaneous control of many reaction parameters, which may translate into the type of obtained phase.

## 2 Experimental Section

### 2.1 Materials

Theobromine was obtained from Sigma-Aldrich. Theophylline (TPH) and pyromellitic acid (benzene-1,2,4,5-tetracarboxylic acid, PMLA) were purchased from TriMen Chemicals. These reagents were used without prior purification (Figure 1).

**Figure 1:**
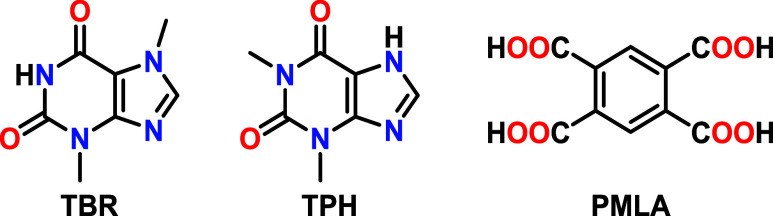
Structures of theobromine (TBR), theophylline (TPH), and benzene-1,2,4,5-tetracarboxylic acid (PMLA).

### 2.2 Cocrystallization Methods

#### 2.2.1 Cocrystallization by Slow Evaporation

Theobromine (TBR) and pyromellitic acid (PMLA) were dissolved in a methanol/water mixture at a 2:1 stoichiometric ratio by heating and stirring. The prepared solution was left for slow evaporation to obtain 2:1 TBR·PMLA single crystals. Theophylline (TPH) and pyromellitic acid (PMLA) in a 2:1 stoichiometric ratio were dissolved by heating in a methanol-nitromethane mixture. After slow evaporation of this solution, single crystals of the TPH·PMLA 2:1 **I** cocrystal were obtained. Polymorph TPH·PMLA 2:1 **II** was similarly obtained using a methanol-chloroform mixture. Single crystals in the form of large blocks of the TPH·PMLA·MeOH 2:1:2 cocrystal solvate were identified for the first time from the cocrystallization of TPH with PMLA in a 4:1 stoichiometric ratio, conducted in a methanol-nitromethane solvent mixture. These crystals were used to nucleate the cocrystallization of TPH with PMLA in a 2:1 stoichiometric ratio in the same solvent mixture, yielding a pure cocrystal solvate. The TPH·PMLA·H_2_O 1:1:2 cocrystal hydrate was obtained by cocrystallization of TPH and PMLA in a 1:1 stoichiometric ratio in an acetonitrile/water mixture, followed by slow evaporation of the solution. The purity of the TBR·PMLA 2:1 (Figure S1), TPH·PMLA 2:1 (polymorphs**I** and **II**), and TPH·PMLA·H_2_O 1:1:2 phases was confirmed by powder analysis (Figure S2). Details regarding the crystallization conditions are presented in Table S1.

#### 2.2.2 Mechanochemical Synthesis

Mechanochemical reactions were carried out using Retsch MM300 oscillatory ball mill in 3 mL stainless steel jars with one 6 mm stainless steel ball placed inside the jar at the constant frequency of 25 Hz. Grindings of TBR or TPH with PMLA in a 2:1 stoichiometric ratio were performed without (neat grinding) or with the addition of a small volume of solvent (methanol, acetonitrile, ethyl acetate, chloroform) for 0.5 to 2 h. Neat or water-assisted grindings of TPH and PMLA in a 1:1 stoichiometric ratio were carried out for 1 to 2 h. Details on conditions for cocrystallization by grinding are shown in Tables S2–S4.

#### 2.2.3 Microwave-Assisted Slurry Cocrystallization

Microwave-assisted cocrystallizations were carried out using an Anton Paar Monowave 200 microwave reactor. Stoichiometric amounts of theobromine or theophylline and pyromellitic acid, a magnetic stirrer, and 200 μL of the selected solvent (water, methanol, acetonitrile, ethyl acetate) were introduced into a 10 mL borosilicate glass vial. The vial was tightly closed and placed in a microwave reactor. After reaching the set temperature, the reaction was conducted for 5 or 10 min while stirring the suspension continuously at 600 rpm. The apparatus software automatically adjusted its microwave power to the sample during the experiment. After the process was completed, the vials were opened, and the solvent was allowed to evaporate. The obtained solids were characterized by powder X-ray diffraction. Details on the microwave-assisted cocrystallization conditions are shown in Tables S5–S7.

### 2.3 Low-Temperature Single-Crystal X-ray Diffraction Study

Reflections intensity data for single crystals of the described PMLA multicomponent systems were collected at 134 K using an Oxford Diffraction SuperNova diffractometer equipped with monochromatic Cu *K*α radiation (λ = 1.54184 Å) and Oxford Instruments Cryosystem cold nitrogen-gas blower. CrysAlisPRO software was used for data collection and data reduction.^40^ Crystal structures of TBR·PMLA 2:1, both TPH·PMLA 2:1 polymorphs and TPH·PMLA·H_2_O 1:1:2 were solved using ShelXT 2018/2.^41^ In turn, for the TPH·PMLA·MeOH 2:1:2 structure solution, ShelXS was applied.^42^ Refinement was performed using the full-matrix least-squares method on *F* ^2^ with SHELXL 2018/3.^43^ The Olex2 program was used as an interface for structure solution, refinement, and structural analysis.^44^ The relevant crystallographic data and refinement details are listed in Table S8. Nonhydrogen atoms were refined with anisotropic displacement parameters. Positions of hydrogen atoms were derived from difference Fourier maps and refined freely. The carboxylic groups of pyromellitic acid molecules in TPH·PMLA·MeOH 2:1:2 are disordered over two positions with fixed occupancies of 50%. The solvent molecule in the obtained cocrystal solvate could not be modeled satisfactorily and their contribution to the structure factors was eliminated using the solvent mask command in Olex2. The calculated void volume of 429.4 Å^3^, occupied by the solvent, corresponds to 14.8% of the cell volume (Figure S3), and the determined number of electrons per free space is 144.8, corresponding to 8 molecules of methanol per unit cell. CCDC 2427610–2427614 contain the supplementary crystallographic data for this paper. This data can be obtained free of charge via www.ccdc.cam.ac.uk/data_request/cif, or by emailing data_request@ccdc.cam.ac.uk, or by contacting The Cambridge Crystallographic Data Centre, 12 Union Road, Cambridge CB2 1EZ, UK; fax: + 44 1223 336033.

### 2.4 Variable-Temperature Single-Crystal X-ray Diffraction Study

A high-quality block-shaped single crystal of TPH·PMLA·MeOH 2:1:2 with dimensions of 0.32 × 0.38 × 0.43 mm^3^ was mounted on a round 0.4 mm loop using grease and utilized for variable-temperature single-crystal X-ray diffraction studies. Reflection intensities were collected from 300 to 425 K, with 10 K intervals within the 340–410 K temperature range. The single-crystal experiments were conducted on a New Xcalibur EosS2 diffractometer using graphite-monochromated Cu *K*α radiation (λ = 1.54184 Å). The crystal was maintained under a nitrogen stream, and the temperature during data collection was regulated using an Oxford Instruments CryoJet-HT device. Data were collected and processed using CrysAlisPRO.^40^ The crystal structure was solved using the intrinsic phasing method with SHELXT and refined using full-matrix least-squares on *F* ^2^ with SHELXL within the Olex2 package.^41,43,44^ All nonhydrogen atoms were refined with anisotropic displacement parameters. The hydrogen atoms were placed using calculated positions and refined as riding on their parent atoms. Both carboxylic groups of PMLA are disordered over two positions, with fixed occupancies at 50%. Void volume and the electron number per void in the unit cell were calculated using solvent masking in Olex2.^44^ CCDC 2435404–2435414 contain the supplementary crystallographic data for this paper. This data can be obtained free of charge via www.ccdc.cam.ac.uk/data_request/cif, by emailing data_request@ccdc.cam.ac.uk, or by contacting The Cambridge Crystallographic Data Centre, 12 Union Road, Cambridge CB2 1EZ, UK; fax: + 44 1223 336033.

### 2.5 Powder X-ray Diffraction (PXRD)

The powder X-ray diffraction patterns for the TBR-PMLA and TPH-PMLA phases were recorded in the angular range of 4 ≤ 2θ ≤ 50°, with a 0.05° step between thetas and a 1s time per step using monochromatic Cu *K*α_1_ (1.54056 Å) radiation with a Bruker AXS D8 ADVANCE powder diffractometer. Based on the solved crystal structures, theoretical powder patterns were generated using Mercury software.^45^ KDif software was utilized to analyze and compare the experimental PXRD patterns for multicomponent systems obtained from grinding or using microwave radiation with theoretical powder patterns derived from the crystal structures.^46^

### 2.6 Steady-State Absorption Spectroscopy

The powdered samples of the obtained cocrystals were dissolved in Millipore quality (MQ) distilled water (18 MΩ cm) and analyzed using a Cary 100 (Agilent) dual-beam UV–vis spectrometer. UV–vis absorption spectra were recorded in the 200–400 nm range with 1 nm increments. Quartz cells with a 2 mm optical length were used. Standard curves were prepared from solutions with known concentrations. Plots of substance concentration versus absorbance at the detection wavelength (Figure S4) were generated. A linear relationship was obtained, and the slope was calculated from the graph. To determine the cocrystals’ solubility, saturated aqueous solutions of each were prepared, and then 50 μL of saturated solution was aspirated from the decanted liquid and filled to 1 mL once again with MQ water. The absorbance at the detection wavelength (λ_det_) was measured, and the concentration of the substance was determined by using the Beer–Lambert equation. All experiments were repeated five times at room temperature (approximately 25 °C), and the average of the results has been presented with double standard deviation. Magnetic stirring was used to ensure complete dissolution.

### 2.7 Simultaneous Thermal Analysis

The thermal properties of the samples were analyzed using a PerkinElmer STA 6000 Simultaneous Thermal Analyzer. A sample, approximately 10 mg, was heated from room temperature to 500 °C, under a nitrogen purge (20 °C min^–1^) at a rate of 10 °C min^–1^. Weight loss was monitored as a function of the temperature.

### 2.8 Biological Studies

The antibacterial properties of the compounds were tested against selected bacteria: *Micrococcus luteus*, *Bacillus subtilis*, *Pseudomonas fluorescens*, and *Escherichia coli*. The antifungal activity of the compounds was tested against *Fusarium culmorum*, *Fusarium graminearum*, *Trichoderma atroviridae*, *Trichoderma harzianum*, *Alternaria alternata*, and *Botrytis cinerea*. All cultures of microorganisms were obtained from the collection of Pure Cultures of the Facility of Microbiology of the Department of Soil Science and Microbiology of the Poznan University of Life Sciences. The well-diffusion method was used to assess the antimicrobial properties of the compounds. Broth medium was used for bacterial tests, while potato dextrose agar (PDA) was used for the cultivation of mold fungi. 6 mL each of liquidized medium was poured into sterile Petri dishes and allowed to solidify. Next, two sterile glass rings with a diameter of 0.5 cm were placed on the surface of each plate. Then, 20 mL each of liquid medium containing suspensions of the tested microorganisms was introduced. The final suspension of bacteria had a density of 10^7^ cells/cm^3^, obtained from 48 h cultures on broth slants, and fungal suspension had a density of 10^8^ spores/cm^3^, obtained from 5-day cultures on PDA slants. After the medium solidified, the glass rings were removed with a pencil, obtaining two wells on each plate. 0.1 mL of compound dissolved in pure dimethyl sulfoxide was introduced into one well, and 0.1 mL of pure dimethyl sulfoxide was introduced into the other well, which was used as a control. Each compound was tested in quadruplicate. The plates were incubated for 48 h in a thermostat at 27 °C for *M. luteus*, *B. subtilis*, and *P. fluorescens* cultures, and the *E. coli* culture at 37 °C. All fungal cultures were incubated for 5 days in a thermostat at 24 °C. At the end of the incubation period, the diameters of growth inhibition of the tested strains were measured using calipers.

## 3 Results and Discussion

In this article, three techniques of cocrystallization were used to obtain cocrystals of purine alkaloids (TBR and TPH) with benzene-1,2,4,5-tetracarboxylic acid (PMLA). The use of green chemical methods made it possible to significantly shorten the process of obtaining cocrystals compared to the slow evaporation of the solution.

Cocrystallizations by grinding and microwave techniques were performed multiple times, not only to produce more material for further analyses but also to test the reproducibility of the designed syntheses. Mechanochemical cocrystallizations with purine alkaloids were conducted in 3 mL jars. It was possible to scale the syntheses using TPH from 20 to 100 mg of total substrate mass. For theobromine, the total mass of components could not exceed 50 mg. Microwave cocrystallizations were performed in 10 mL vials. Theobromine synthesis was scalable within the range 30–70 mg of total substrate mass. In comparison, for theophylline, cocrystallizations used a total substrate mass between 40 and 55 mg. No experiments were performed on a scale larger than 100 mg, which could be the next step in exploring the potential for preparing the described multicomponent systems on a larger scale.

### 3.1 Synthetic Route

Neat grinding of TBR with PMLA in a 2:1 stoichiometric ratio resulted in the formation of the TBR·PMLA 2:1 cocrystals within 30 min. Regardless of the solvent used for liquid-assisted grinding, the TBR·PMLA 2:1 cocrystal is also formed with the conversion of the substrates depending on the reaction time (Figure 2). Grinding assisted by methanol, acetonitrile, and ethyl acetate were carried out for 30 min. In turn, after 30 min of the water- or chloroform-assisted grinding, no full conversion of the substrates was observed, which required a doubling of the grinding time.

**Figure 2:**
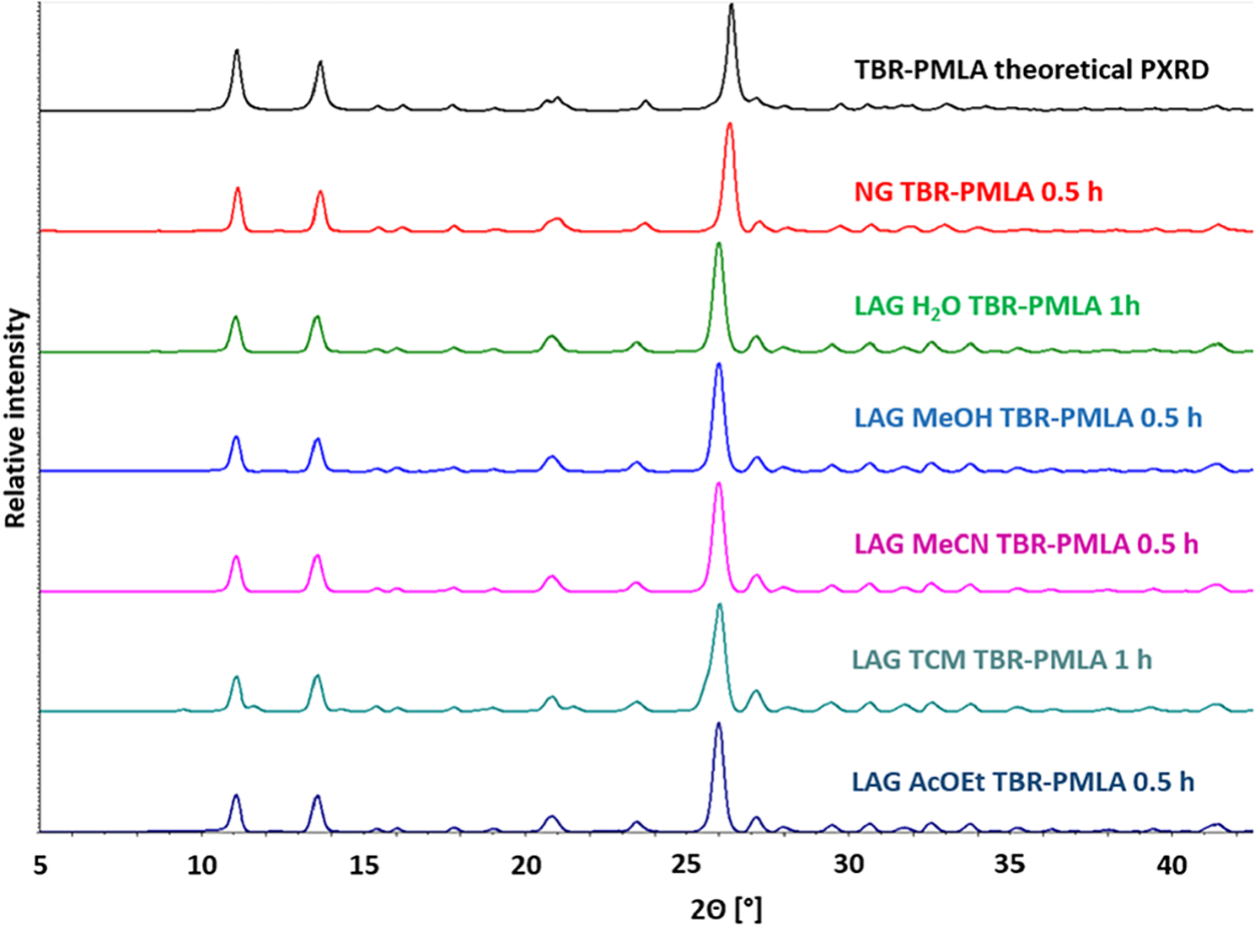
Comparison of theoretical powder diffractogram of TBR·PMLA 2:1 with powder patterns for solids obtained by neat or liquid-assisted grinding of TBR and PMLA in a 2:1 stoichiometric ratio.

Microwave cocrystallizations involving theobromine were performed using different solvents for 5 to 10 min at temperatures ranging from 100 to 175 °C (Table S5). The use of water enabled the formation of the TBR·PMLA 2:1 cocrystal during a microwave reaction conducted at 125 °C for 10 min (Figure S7). Increasing the temperature did not negatively affect the formation of the cocrystal. The least beneficial was the use of methanol for this process. At 100, 125, and 150 °C, the substrates did not fully convert to the 2:1 TBR·PMLA cocrystal, and raising the temperature to 175 °C resulted in the inhibition of the cocrystallization process (Figure S8). Practically full conversion of the substrates to the TBR·PMLA 2:1 cocrystal was observed during the process conducted in acetonitrile or ethyl acetate at 150 °C for 10 min (Figure 3). Lower temperatures did not lead to full conversion of the substrates.

**Figure 3:**
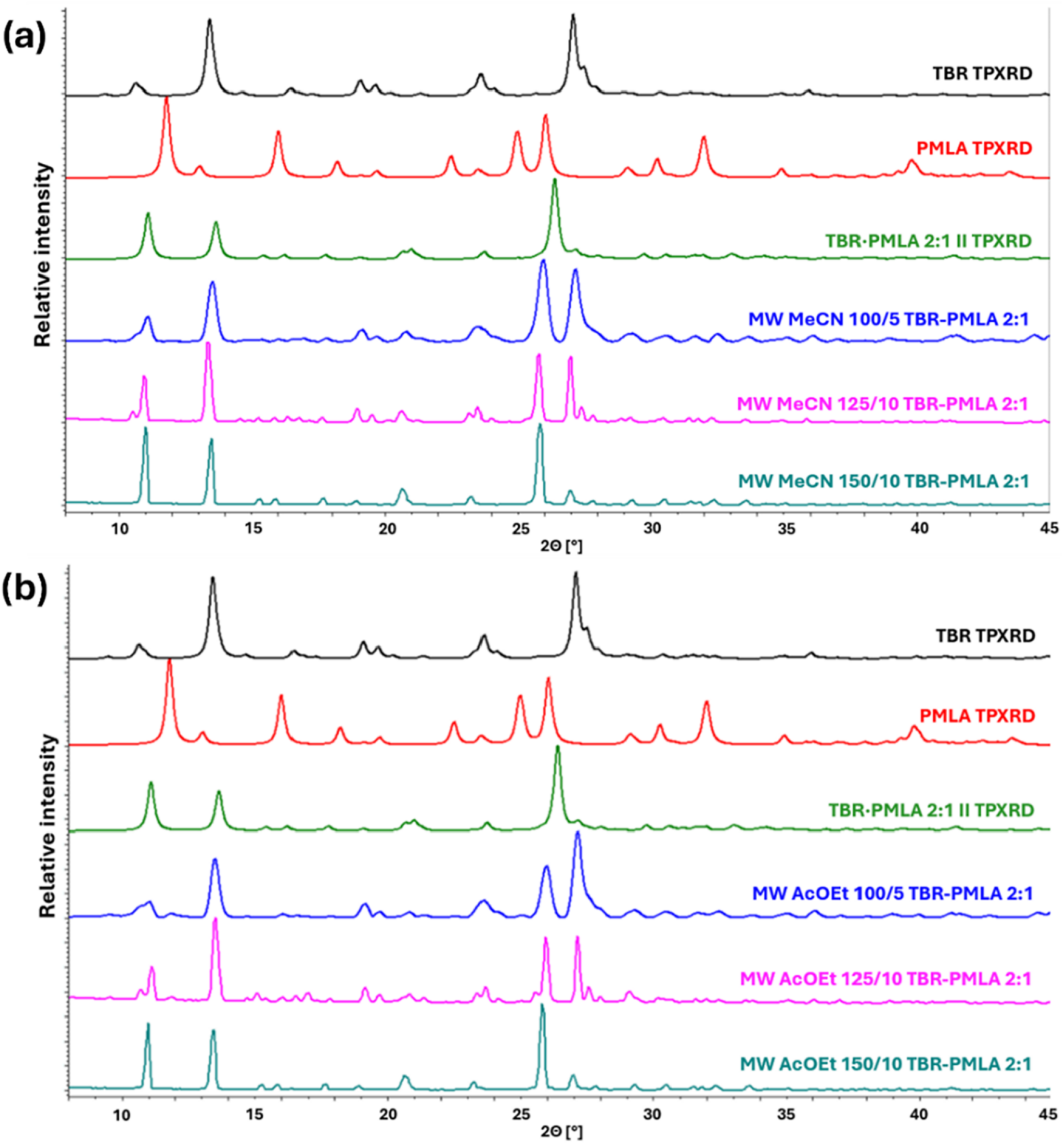
Comparison of theoretical powder diffractograms of TBR, PMLA, and TBR·PMLA 2:1 with powder patterns for solids obtained by microwave-assisted slurry cocrystallization of TBR and PMLA in a 2:1 stoichiometric ratio performed in acetonitrile (a) or ethyl acetate (b).

Selection of appropriate cocrystallization conditions facilitated the targeted preparation of distinct forms of the TPH-PMLA multicomponent systems. The choice of solvent in each technique was critically important. Cocrystallization of theophylline with pyromellitic acid in a stoichiometric ratio of 2:1 from a methanol/nitromethane mixture yielded polymorph **I** of TPH·PMLA 2:1. Substituting nitromethane with chloroform resulted in polymorph **II** formation. The TPH·PMLA·H_2_O 1:1:2 cocrystal hydrate was successfully obtained through cocrystallization of TPH and PMLA in a 1:1 stoichiometric ratio from an acetonitrile–water system (Figure S2 and Table S1).

Neat or chloroform-assisted grinding for 30 min resulted in the formation of TPH·PMLA 2:1 form **I** (Figure 4a). Grinding in the presence of methanol or acetonitrile required a reaction time of 60 min to obtain this form. The TPH·PMLA·MeOH 2:1:2 phase was not observed as a product of methanol-assisted grinding. Milling with ethyl acetate required 2 h to obtain polymorph **I**. Powder diffraction patterns for solids obtained from grinding with ethyl acetate or acetonitrile indicated that polymorph **II** of TPH·PMLA 2:1 forms during grinding conducted in a shorter time, while its extension leads to polymorph **I** formation (Figures S5 and S6). This proves that the grinding of TPH with PMLA in a 2:1 stoichiometric ratio is a stepwise process, and polymorph **II**, formed first, is a kinetic product, while polymorph **I** is a thermodynamic product as the final product of the mechanochemical reaction.

**Figure 4:**
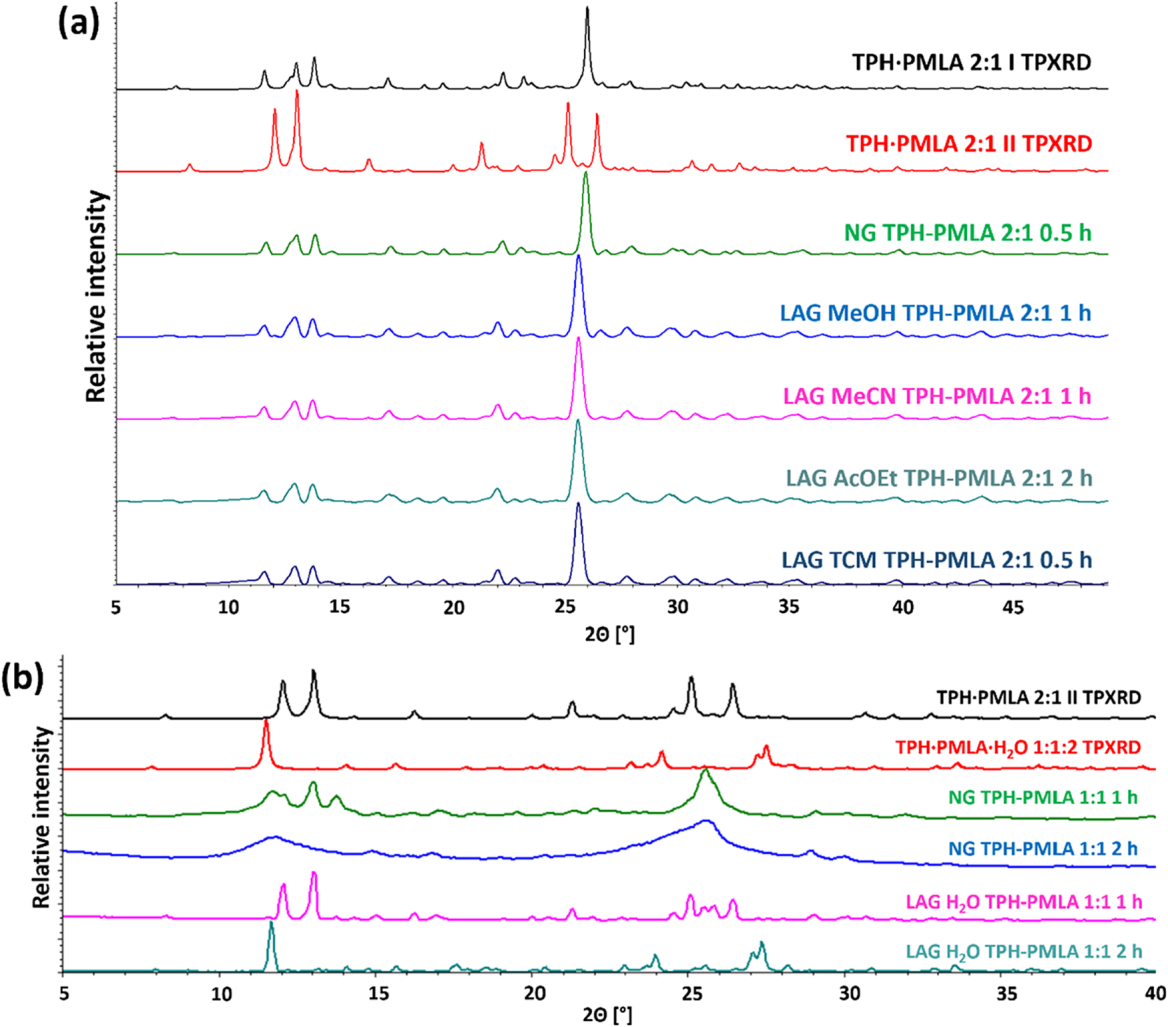
Powder analysis for TPH and PMLA cocrystallization processes by grinding. (a) Comparison of theoretical powder diffractograms for both forms TPH·PMLA 2:1 **I** and **II** with powder patterns for solids obtained by neat or liquid-assisted grinding of TPH and PMLA in a 2:1 stoichiometric ratio. (b) Comparison of theoretical powder diffractograms for TPH·PMLA 2:1 **II** and TPH·PMLA·H_2_O 1:1:2 with powder patterns for solids obtained by neat or water-assisted grinding of TPH and PMLA in a 1:1 stoichiometric ratio.

The formation of the TPH·PMLA·H_2_O 1:1:2 cocrystal hydrate was confirmed by powder X-ray diffraction analysis of the solid obtained through water-assisted grinding of TPH and PMLA in a 1:1 stoichiometric ratio (Figure 4b) conducted for 2 h. Milling under the same conditions for 1 h yielded TPH·PMLA 2:1 form **II**, but not in the pure form. Neat grinding of TPH and PMLA in a 1:1 stoichiometric ratio, regardless of reaction time, leads to amorphous phase formation.

Microwave-assisted cocrystallization for the TPH-PMLA 2:1 system carried out for 5 min using various solvents at different temperatures (Table S6) also allowed for the controlled formation of a specific polymorphic form. This is particularly visible when water is used as the liquid medium (Figure 5a). At 75 °C, polymorph **II** is formed, while at higher temperatures, polymorph I is formed. In the presence of methanol, acetonitrile, or ethyl acetate (Figures S9–S11), regardless of the process temperature, polymorph **II** is selectively formed. During the microwave-assisted slurry cocrystallization experiments performed in methanol, no TPH·PMLA·MeOH 2:1:2 phase was observed (Figure S9). The TPH·PMLA·H_2_O 1:1:2 phase is formed during microwave-assisted cocrystallization in a 1:1 stoichiometric ratio of substrates (Table S7) in water as the liquid medium, regardless of the temperature process (Figure 5b). For the TPH-PMLA 1:1 system, when using solvents other than water (methanol, acetonitrile, ethyl acetate), regardless of the process temperature, the TPH·PMLA 2:1 **II** phase is formed (Figures S12–S14), but not in the pure form (the reflections originating from unreacted substrates are observed).

**Figure 5:**
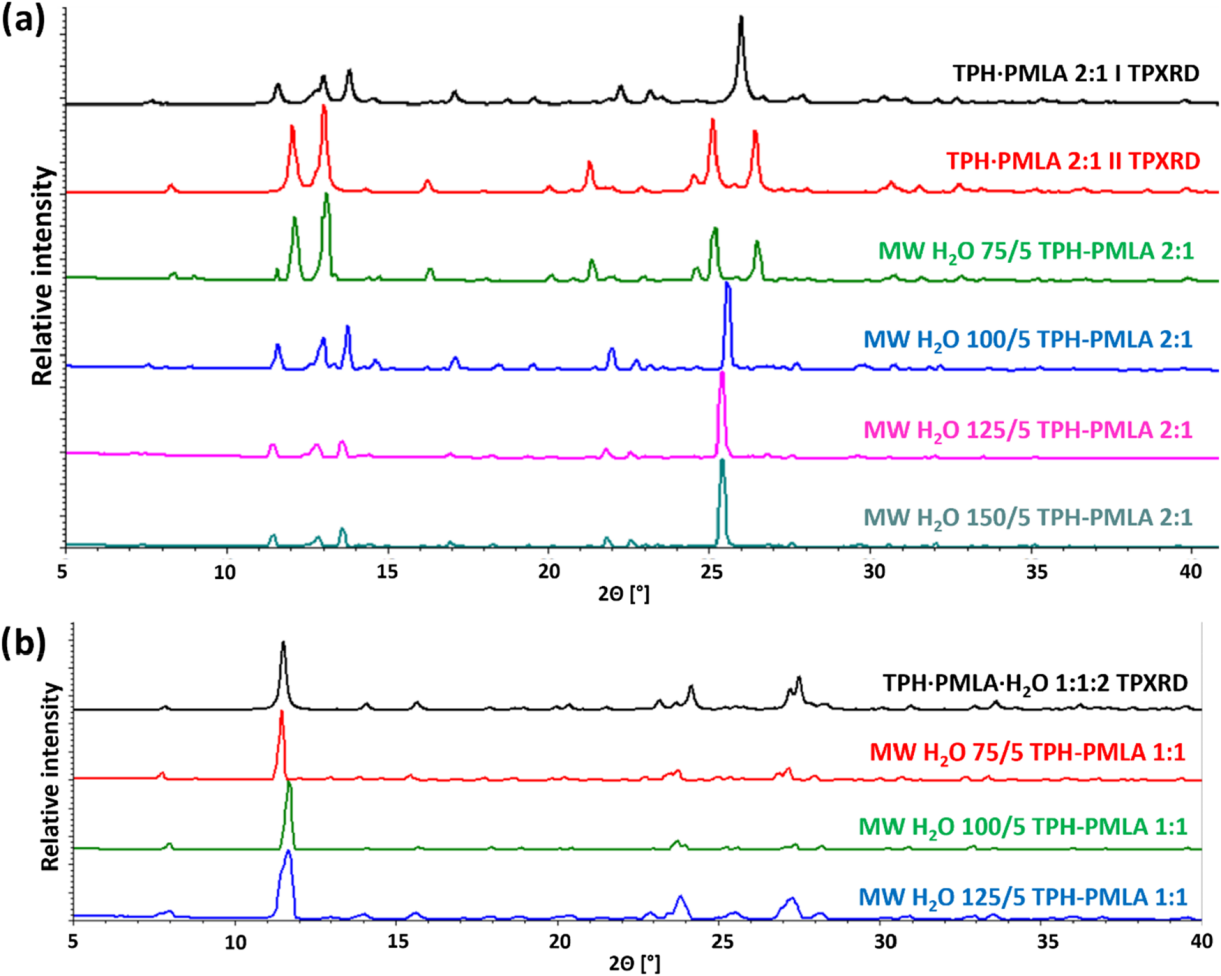
Powder analysis of microwave-assisted cocrystallization processes of TPH and PMLA conducted in water. (a) Comparison of theoretical powder patterns of both forms (**I** and **II**) of TPH·PMLA 2:1 cocrystal with powder patterns for solids obtained by microwave-assisted cocrystallization of TPH and PMLA in a 2:1 stoichiometric ratio performed in water. (b) Comparison of theoretical powder diffractogram of TPH·PMLA·H_2_O 1:1:2 with powder patterns for solids obtained by microwave-assisted cocrystallization of TPH and PMLA in a 1:1 stoichiometric ratio performed in water.

### 3.2 Single-Crystal Structure Description

Theobromine and pyromellitic acid formed a cocrystal in a 2:1 stoichiometric ratio (Figure S15), which crystallized in the triclinic *P*1̅ space group. In the TBR·PMLA 2:1 crystal lattice, all of the PMLA molecules are hydrogen-bonded to four purine alkaloids (Figure 6). The absence of proton transfer between coformers and cocrystal formation is confirmed by the presence of COOH···N_imidazole_ heterosynthons (O2–H2···N4 hydrogen bonds, Table S9). Consistent with previous studies, TBR molecules in the presence of a benzoic acid derivative as a coformer preferentially form an amide–amide homosynthon *R*_2_^2^(8) through the C=O­(*exo*-carbonyl)···H–N­(pyrimidine) hydrogen bond involving the *exo*-carbonyl oxygen atom (N1–H1···O5^ii^ hydrogen bonds), which are observed in the described structure. Simultaneously, the second carbonyl group of the TBR molecule forms noncovalent O–H­(carboxyl)···O=C­(*endo-*carbonyl) interactions with carboxyl groups of PMLA (O4–H4···O6^i^ hydrogen bonds). Components of this cocrystal, sustained by the aforementioned hydrogen bonds, form layers parallel to the (113̅) plane (Figure 6).

**Figure 6:**
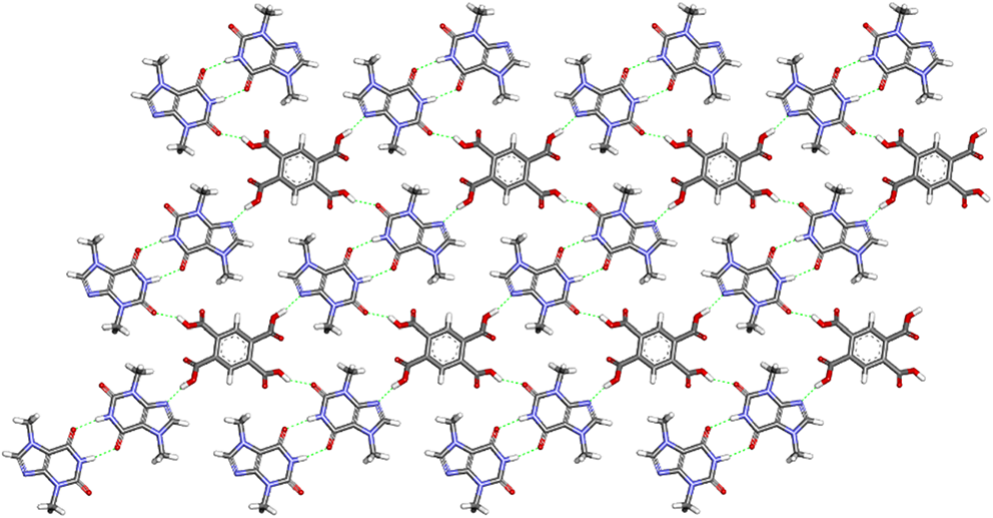
Noncovalent interactions between theobromine and pyromellitic acid molecules in the TBR·PMLA 2:1 crystal within the layer parallel to the (113̅) plane.

Theophylline and pyromellitic acid in a 2:1 stoichiometric ratio, depending on the cocrystallization conditions, form two polymorphic TPH·PMLA 2:1 forms **I** and **II** (Figures S16 and S17), differing in the arrangement of the molecules in the crystal lattice. In form **I**, the components are arranged into layers parallel to the (131̅) plane (Figure 7a). Two opposite carboxyl groups of PMLA are noncovalently bonded to the imidazole nitrogen atoms of TPH molecules through COOH···N_imidazole_ interactions (O2–H2···N4 and O6–H6···N4A hydrogen bonds, Figures 7a, S16, and Table S10). Alkaloid molecules form two independent centrosymmetric homosynthons, *R*_2_^2^(10) involving *exo*-carbonyl oxygen atoms via N–H···O hydrogen bonds (one of them through N3–H3B···O9^iv^ and the other through N3A-H3A···O9A^ii^ interactions, Table S10). The carboxylic groups of PMLA molecules are hydrogen-bonded via O8–H8···O5 interactions to form cyclic centrosymmetric *R*_2_^2^(14) homosynthons (Figure 7a). Additionally, an O4–H4···O10 hydrogen bond between one of the carboxyl groups of PMLA and the *endo*-carbonyl oxygen atom of one of the TPH molecules is also observed.

**Figure 7:**
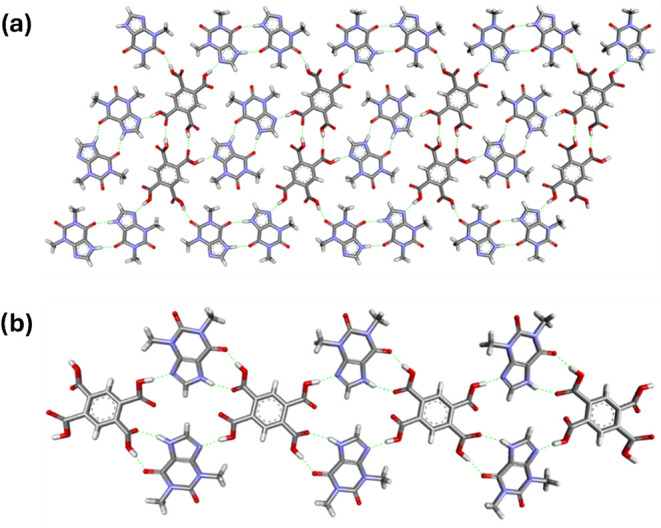
(a) 2D structure of TPH·PMLA 2:1 **I** representing noncovalent interactions between cocrystal components within the molecular layer parallel to the (131̅) plane, in a view along [110] direction. (b) Fragment of TPH·PMLA **II** crystal structure representing 1D molecular chain through [011] direction, in a view along the [301] direction.

In contrast to polymorph **I**, no supramolecular homosynthons in TPH·PMLA 2:1 **II** are observed. Similar to form **I**, two opposite carboxyl groups of PMLA in polymorph **II** form COOH···N_imidazole_ heterosynthons with the imidazole nitrogen atoms of theophylline molecules (O2–H2···O4 interactions, Figure S17).

The remaining carboxyl groups form R_2_^2^(9) heterosynthons with TPH molecules through the O4–H4···O5^ii^ and N3–H3···O3^i^ hydrogen bonds (Table S11). Components of TPH·PMLA 2:1 **II**, through the aforementioned hydrogen bonds, are arranged into molecular chains along the [011] direction (Figure 7b). This structural analysis confirms that both TPH·PMLA 2:1 polymorphs, which crystallize in a triclinic *P*1̅ space group, are examples of synthon polymorphism.^47^

The hydrogen bond analysis in the TPH·PMLA·MeOH 2:1:2 (Figure S18) indicates the formation of analogous hydrogen bond motifs between theophylline and pyromellitic acid molecules as in polymorph **II** of TPH·PMLA 2:1 (Figures 7b and 8a). The formation of COOH···N_imidazole_ (O2–H2···N4 hydrogen bond) and TPH–acid (O4–H4···O5 and N3–H3···O3 hydrogen bonds) heterosynthons is also observed (Figure 8a and Table S12). Minor variations in the conformation of PMLA carboxyl groups are observed (Figure S19). The rearrangement of TPH molecules relative to these groups leads to the formation of channels along the [001] direction which incorporate solvent molecules (Figures 8b and S3).

**Figure 8:**
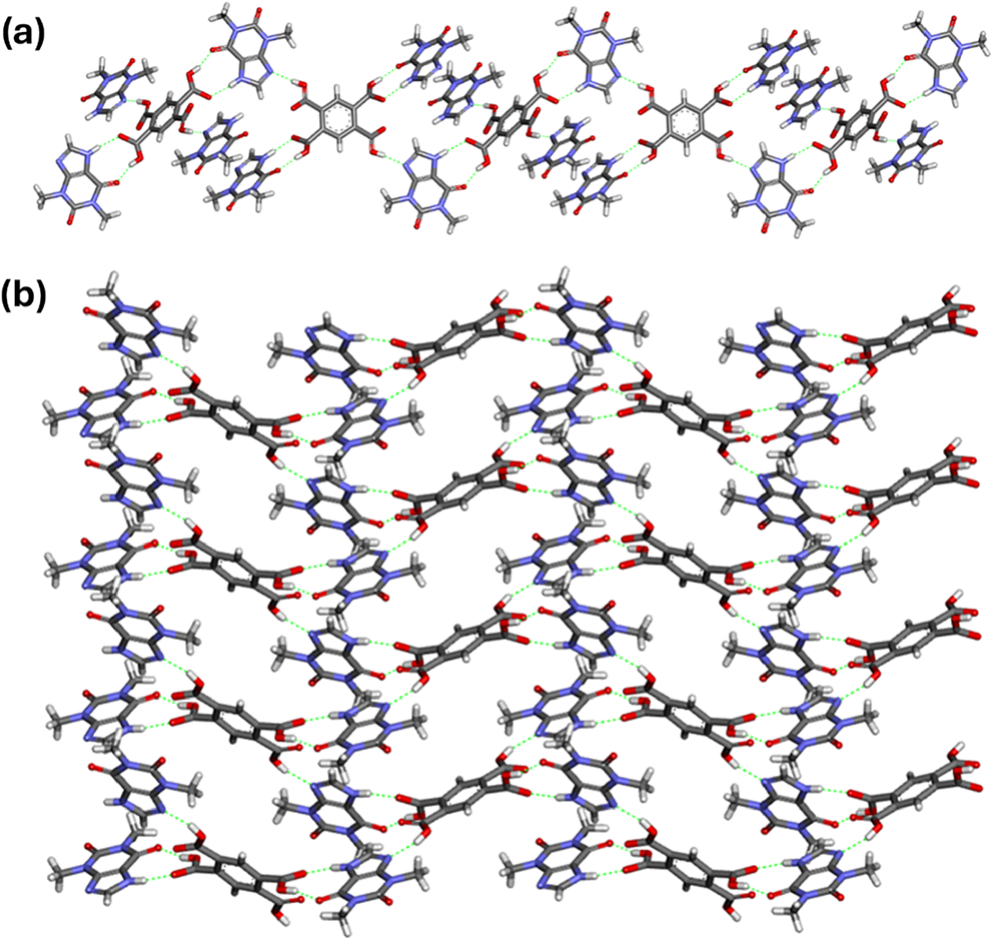
(a) Arrangement of the molecules in the crystal lattice of TPH·PMLA·MeOH 2:1:2 along the [111] direction. (b) Fragment of TPH·PMLA·MeOH 2:1:2 crystal structure in a view along the [105̅] direction.

Screening experiments also showed TPH·PMLA·H_2_O 1:1:2 cocrystal dihydrate formation, which crystallizes in the orthorhombic *Pca*2_1_ space group (Figure S20). In the crystal lattice of this system, theophylline molecules form noncovalent bonds with three pyromellitic acid molecules. The formation of COOH···N_imidazole_ heterosynthon (O2–H2···N4 hydrogen bond), TPH-ACID heterosynthon *R*_2_^2^(9) involving the *exo*-carbonyl oxygen atom of theophylline (O8–H8···O9^iii^ and N3–H3A···O7^i^ hydrogen bonds), and the O4–H4···O10^ii^ interaction between the *endo*-carbonyl oxygen atom of TPH as an acceptor to one of the carboxyl groups of PMLA acid are observed (Table S13). Due to these interactions, the molecules are arranged into a two-dimensional rhombic-grid network, the side length of which is 13.4317(2) Å (Figure 9). Through a complex network of hydrogen bonds involving water molecules (Table S13), a three-dimensional crystal structure is formed (Figure S21).

**Figure 9:**
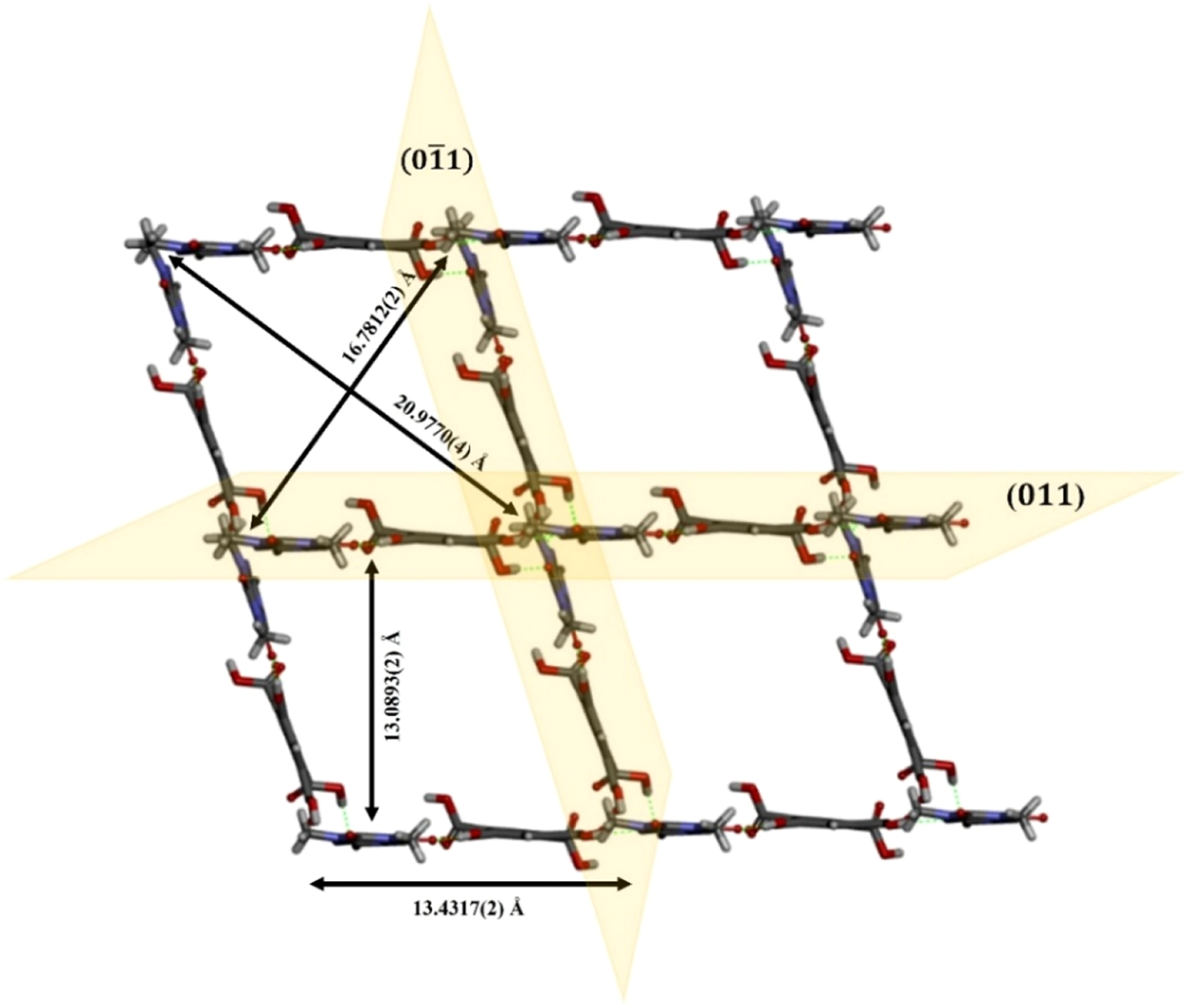
Fragment of 2D rhombic-grid network of TPH·PMLA·H_2_O 1:1:2 (side length: 13.4317(2) Å, diagonals: 16.7812(2) and 20.9770(4) Å) formed by noncovalent interactions between TPH and PMLA molecules without water molecules participation in a view along [100] direction.

### 3.3 Solubility Studies

Solubility studies are an essential part of the research on pharmaceutical cocrystals due to the possibility of enhancing this parameter by using an appropriate coformer for cocrystallization. The selection of benzene-1,2,4,5-tetracarboxylic acid as a coformer (solubility of PMLA in water equals 43.2·10^–3^ mmol·mL^–1^ at 25 °C^48^) results in a TBR·PMLA 2:1 cocrystal formation with 4.5-fold higher water solubility than pure theobromine (Table 1). The use of this coformer disrupts the stable amide–amide homosynthons involving the *endo*-carbonyl oxygen atom of the bromine and π···π interactions between alkaloid molecules. The newly formed interactions could affect the change in crystal lattice energy, which in turn influenced the dissolution kinetics and led to an increased solubility. The improvement of this parameter was certainly also influenced by the better solubility of the coformer in water compared to theobromine, which sped up the dissolution of the alkaloid in water and the formation of new intermolecular interactions.

**Table 1:** Absorption Solubility of PMLA Cocrystals in Water^a^

**alkaloid–PMLA**	**alkaloid solubility in water** (10^**–3**^ **mmol mL** ^**–1**^ **)**	**absorption solubility of alkaloid–PMLA system in water** (10^**–3**^ **mmol mL** ^**–1**^ **)**
TBR·PMLA 2:1	1.83 ^49^	8.17 ± 0.42 (×4.46)
TPH·PMLA 2:1 **I**	45.7 ^16^	14.37 ± 1.04 (×0.31)
TPH·PMLA 2:1 **II**		13.69 ± 1.79 (×0.30)
TPH·PMLA·H_2_O 1:1:2		4.83 ± 0.26 (×0.11)

a The increase relative to alkaloid is shown in parentheses.

The use of pyromellitic acid decreases the theophylline solubility in water. Both TPH·PMLA 2:1 **I** and **II** polymorphs show more than 3 times lower solubility in water than TPH. The cocrystal hydrate TPH·PMLA·H_2_O 1:1:2 is 9.5 times less soluble in water compared to theophylline, probably due to the formation of a complex three-dimensional network of strong hydrogen bonds. Due to difficulties with the repeatability of obtaining TPH·PMLA·MeOH 2:1:2 solvate, this system was not subjected to solubility tests.

### 3.4 Thermal Analysis

Theobromine cocrystal, TBR·PMLA 2:1, is stable up to 203 °C. Beyond this temperature, it undergoes two decomposition steps. The DSC curve exhibits three sharp endothermic peaks (Figure 10). The first DSC peak, observed in the 203–249 °C temperature range (9.2%), can be attributed to the partial loss of pyromellitic acid. The second at 311 °C corresponds to the decomposition of theobromine and the residual part of PMLA. The third DSC peak represents the decomposition of the theobromine molecules.^50,51^ Approximately 2% of the initial sample remained as a charred residue above 500 °C.

**Figure 10:**
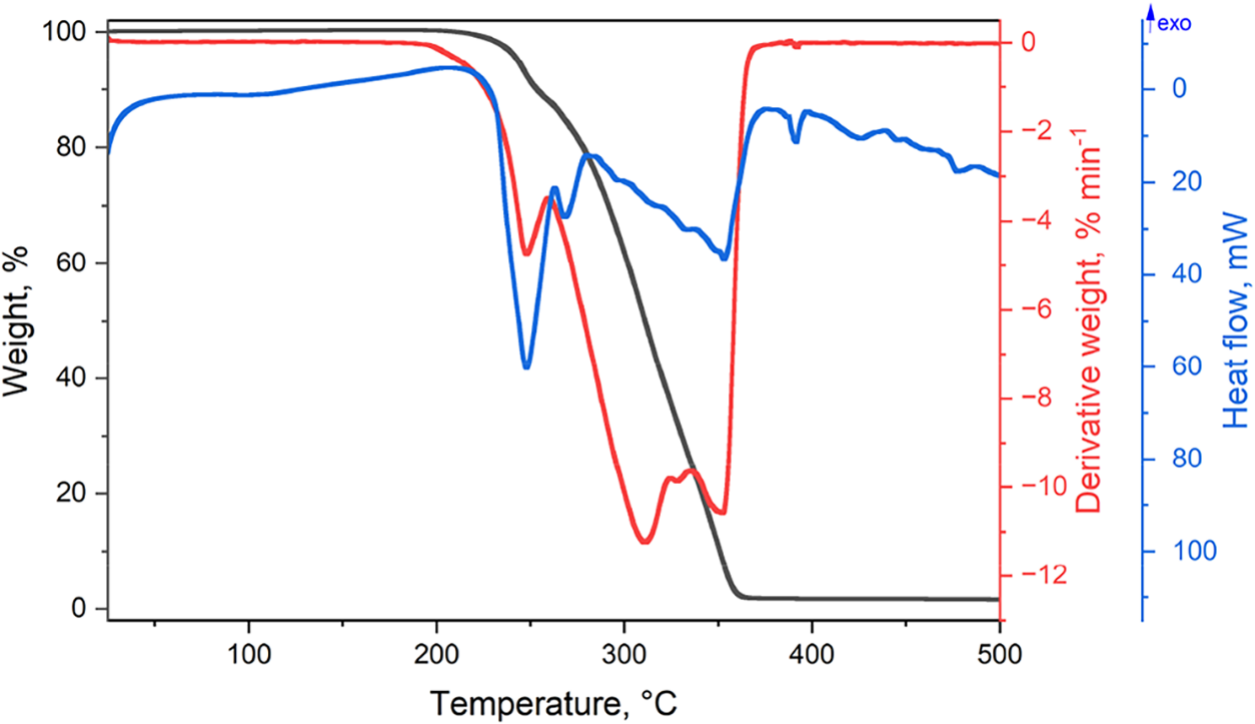
TG (black), DTG (blue), and DSC (red) curves of TBR·PMLA 2:1.

Both TPH·PMLA 2:1 **I** and TPH·PMLA 2:1 **II** are stable up to 205 °C (Figure 11A,B, respectively). The corresponding DSC curves show a double endothermic band for the **I** form and a single endothermic band for the **II** form in the temperature range from 205 to 230 °C. This suggests more complex phase transitions in this cocrystal. Above this temperature, up to 362 °C (for TPH·PMLA 2:1 **I**) and 330 °C (for TPH·PMLA 2:1 **II**), rapid and uniform weight loss is observed. The total weight loss amounts to 56–57%. However, an additional endothermic peak is observed for the **II** form of that loss. The results indicate that the decomposition of theophylline molecules comes first, followed by the gradual decomposition of pyromellitic acid.

**Figure 11:**
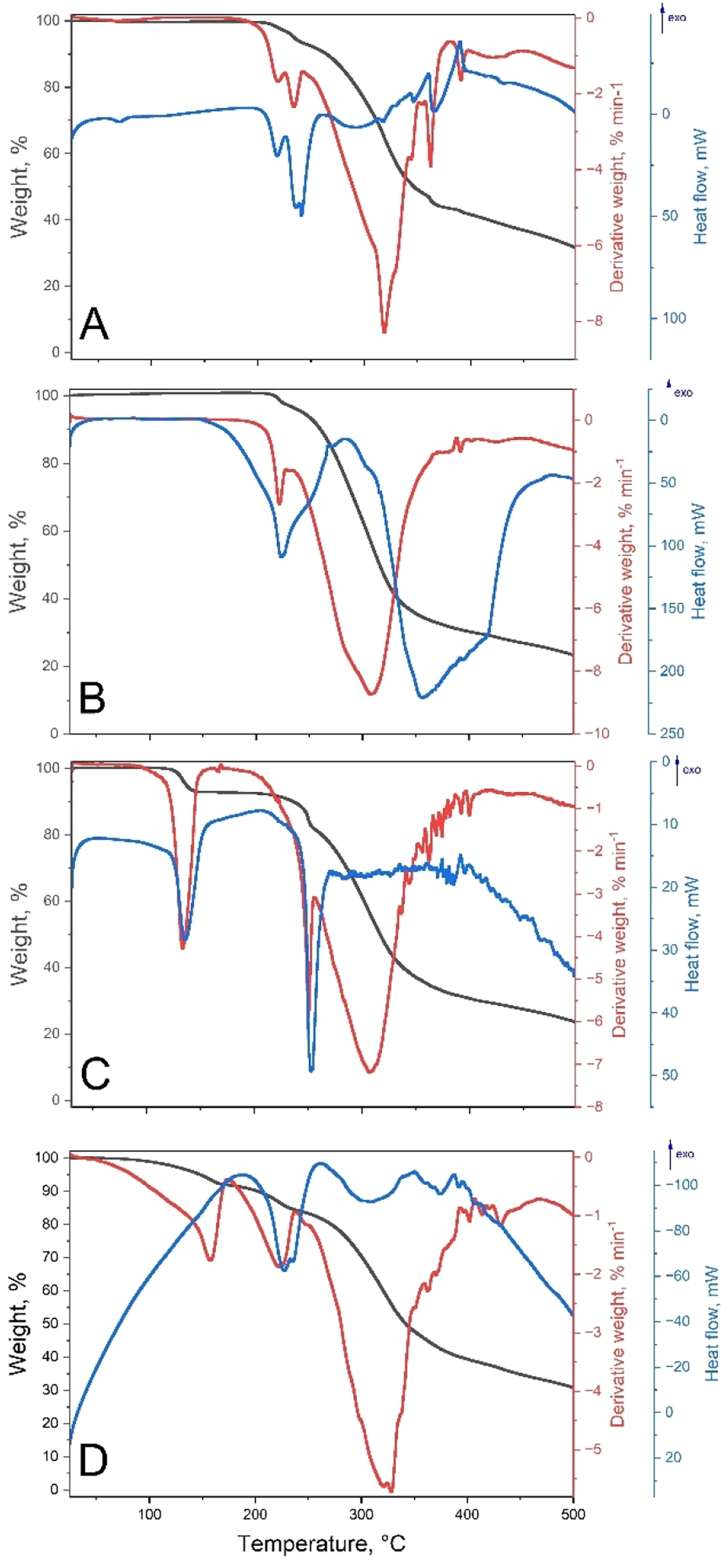
TG (black), DTG (blue), and DSC (red) curves of (A) TPH·PMLA 2:1 **I**, (B) TPH·PMLA 2:1 **II**, (C) TPH·PMLA·H_2_O 1:1:2, and (D) TPH·PMLA·MeOH 2:1:2.

Nonisothermal TG of TPH·PMLA·H_2_O 1:1:2 revealed a weight loss of 7.8% in the 100–200 °C temperature range, equivalent to the stoichiometric water content in this hydrate (Figure 11C). Between 210 and 250 °C, a subsequent weight loss of approximately 12% is observed. Further heating up to 335 °C, a weight loss of about 38% was observed. At 500 °C, the initial sample residue is 23% (the same as for the TPH·PMLA 2:1 **II**, where for the TPH·PMLA 2:1 **I** form, it is 31%). Below 45 °C, the 2:1:2 TPH·PMLA·MeOH remains stable (Figure 11D). The first weight loss is observed in the 45–172 °C temperature range (8.2%), which is caused by the release of methanol molecules. Then, up to 236 °C, the weight loss of about 7.5%. At the temperature of 500 °C, the initial sample residue is 30%, 600 °C–nearly 23%.

### 3.5 VT-SXRD Experiment for TPH·PMLA·MeOH 2:1:2

A variable-temperature single-crystal X-ray diffraction experiment for a single crystal of TPH·PMLA·MeOH 2:1:2 was performed in the 300–415 K temperature range. Crystallographic data and refinement details are included in Tables S14–S16. The main objective was to investigate the solvent release process from the channels formed among components of this cocrystal solvate and how desolvation would affect the behavior of the single crystal. Another aspect was to investigate the effect of the temperature on the thermal expansion of this multicomponent system.

The experiment showed a large biaxial negative thermal expansion and a large uniaxial positive thermal expansion (PTE) of the TPH·PMLA·MeOH 2:1:2 cocrystal solvate. Changes in the value of *a* and *c* parameters indicate negative linear thermal expansion (NTE), which contracts during heating from 300 to 415 K (α_a_ = −355.46 and α_c_ = −95.57 MK^–1^, Figure 12a,c). The expansion of the *b* parameter value is observed and the thermal expansion coefficient (CTE) is α_b_ = 233.40 MK^–1^ (Figure 12b). The linear thermal expansion coefficients (α) were calculated using the standard expression: $\alpha =\frac{1}{L_{0}}\cdot \frac{\mathrm{dL}}{\mathrm{dT}}$ , where *L*_0_ is the initial unit cell parameter at 300 K and $\frac{\mathrm{dL}}{\mathrm{dT}}$ is the rate of change of the lattice dimension with temperature. The values presented above show a colossal negative linear thermal expansion (NLTE) for the *a* parameter and a colossal positive linear thermal expansion for the *b* parameter.

**Figure 12:**
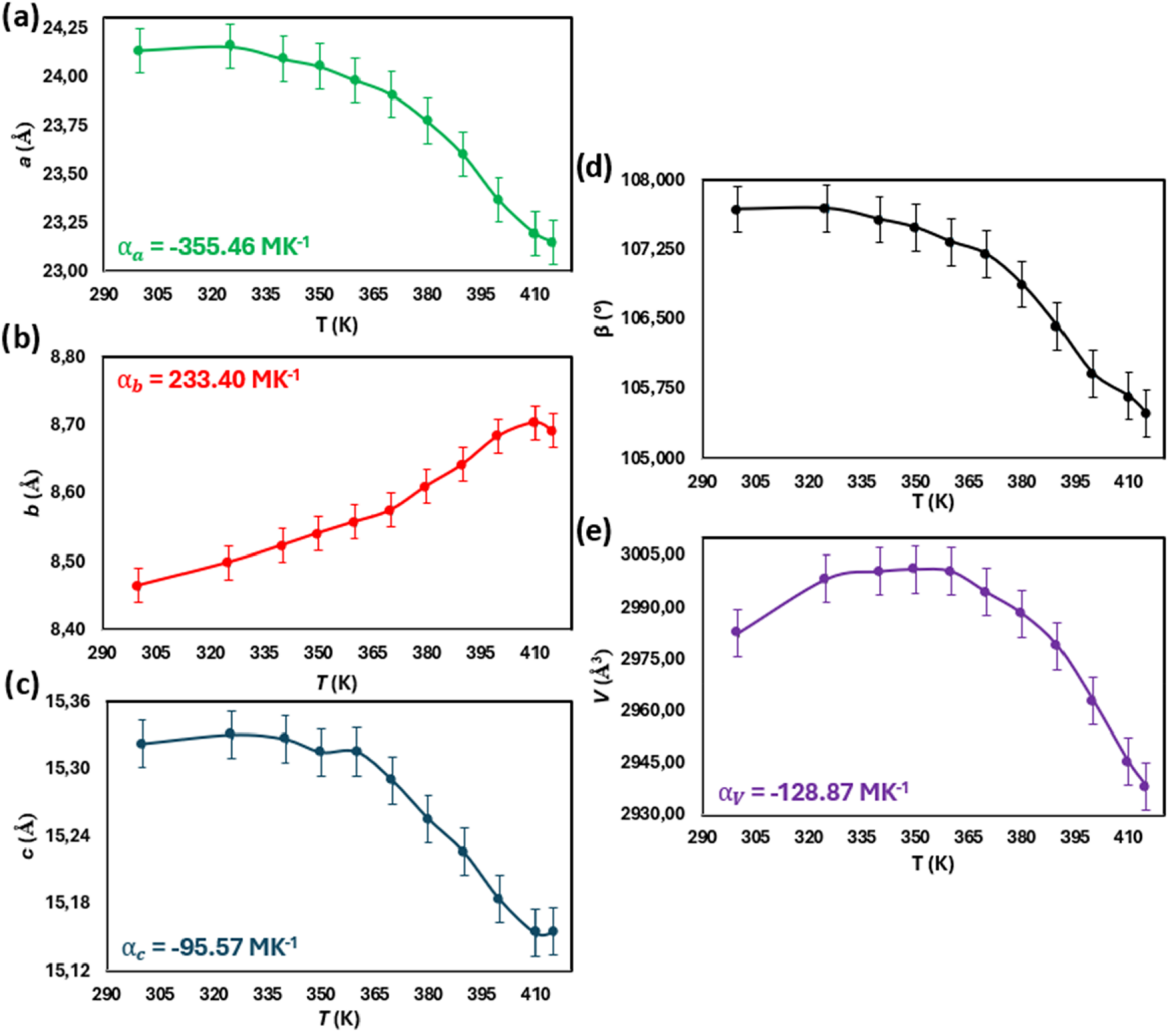
(a–e) Changes in unit cell parameters (*a*, *b*, *c*, and β) and volume (*V*) with increasing temperature in the 300–415 K range.

In addition, a monotonic decrease in the β angle value is observed from 107.679(4)° at 300 K to 105.489(5)° at 415 K (Figure 12d). Interestingly, two stages of volumetric thermal expansion change are observed in the 300–415 K temperature range (Figure 12e). As the temperature rises from 300 to 350 K, a gradual increase in volume is noted from 2982.5(2) Å^3^ to 3001.0(2) Å^3^, and a PTE in this range is characterized by the volumetric thermal expansion coefficient of 131.76 MK^–1^. The switching from PTE to NTE is observed at 350 K (Figure 12e), as a further increase in temperature causes a decrease of volume from 3001.0(2) Å^3^ at 350 K to 2938.3(3) Å^3^ at 415 K. The volumetric CTE determined for this range is −336.04 MK^–1^, indicating an exceptionally large negative volumetric thermal expansion (NVTE). The determined volumetric thermal expansion coefficient parameter in the entire temperature range from 300 to 415 K is −128.87 MK^–1^.

The above data provide insight into the behavior of the single crystal with increasing temperature and determine the directions along which it expands or contracts. However, this does not provide a complete picture because the principal expansion axes do not necessarily coincide with the unit cell axes. The indices of thermal expansion axes with their CTE values were determined using the publicly available PASCAL tool.^52^ Calculations were carried out for the full temperature range (300–415 K) and for two temperature ranges, where an increase (300–350) and then a decrease (350–415 K) of the unit cell volume were observed (Tables S17 and S18). In the full temperature range of 300 and 415 K, the thermal expansion coefficients along the principal axes, *X*1 [0.3424, 0, 0.9396], *X*2 [0.8888, 0, −0.4583], and *X*3 [0, 1, 0] are 43.90, −423.62, and 253.49 MK^–1^, respectively, and −135.09 MK^–1^ is the value of volumetric thermal expansion coefficient. It is worth noting that in the 350–415 K range, where only the decrease in volume is observed, as indicated by the colossal NTE of −336.04 MK^–1^, the indices of the thermal expansion axes practically coincide with the indices determined in the whole 300–415 K temperature range. The determined CTE values along these axes, namely, *X*1 [0.8577, 0, −0.5141], *X*2 [0.363, 0, 0.9318], and *X*3 [0, −1, 0], are −696.55, 61.88, 310.83 MK^–1^, respectively. In both discussed temperature ranges, colossal PTE and colossal NTE values corresponding to the same thermal expansion axes are observed.

The dominant interaction observed along the [010] direction is the π­(TPH)···π­(TPH) interaction between the imidazole fragment of one alkaloid molecule and the pyrimidine fragment of the other. The observed increase in the distance between the centroids of these rings (Figure 13a) aligns with the positive value of the thermal expansion coefficient along the *X*3 axis.

**Figure 13:**
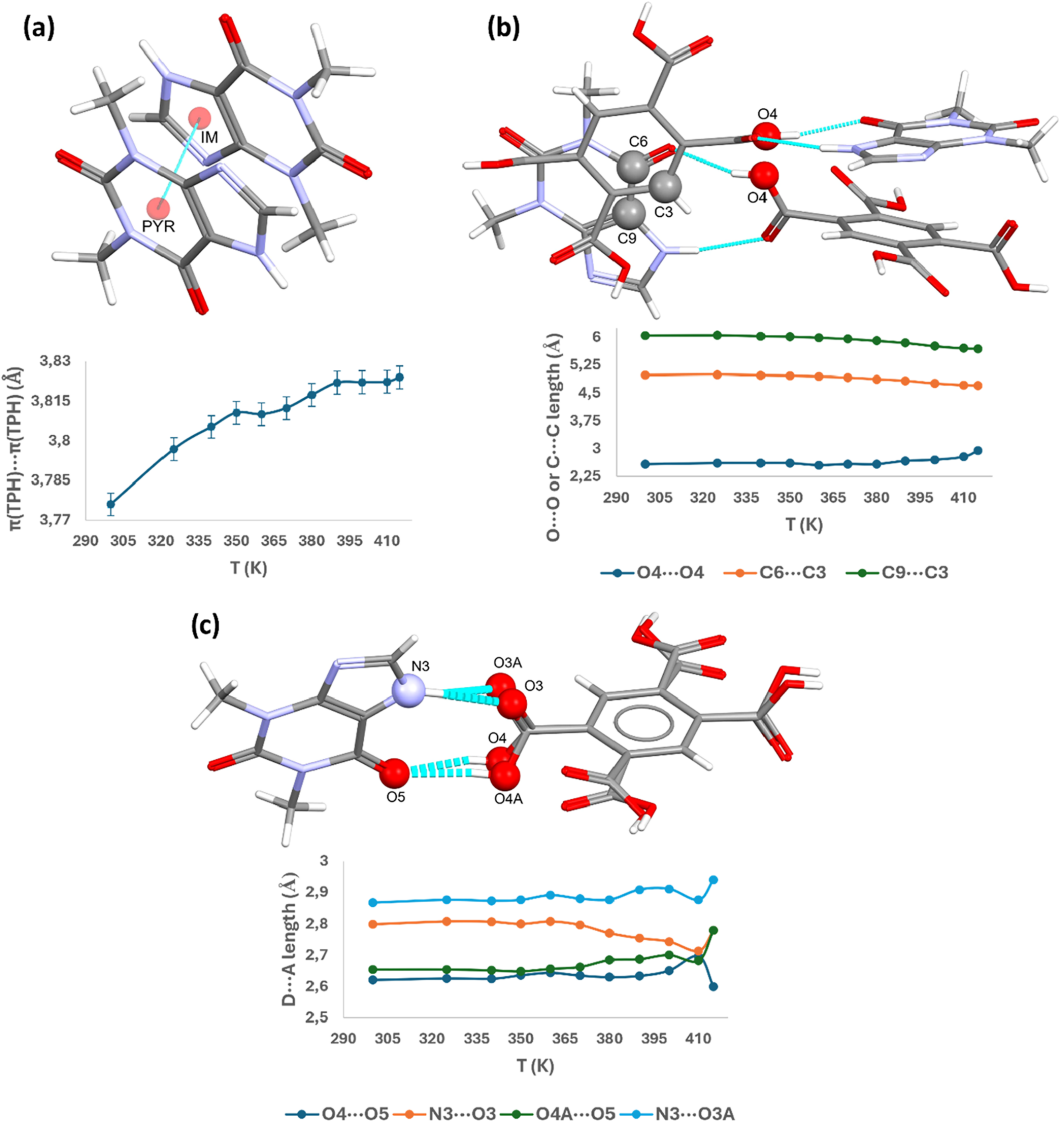
Changes of selected parameters in the TPH·PMLA·MeOH crystal lattice as a function of the temperature. (a) π···π interaction, as the distance between ring centroids, related to the *X*3 [0, 1, 0] direction; (b) distances between O4···O4, C6···C3 and C9···C3 atoms, whose changes relate to the approximate axis [201̅] of thermal expansion; (c) changes in D···A parameter within the TPH-ACID heterosynthon, the hydrogen bonds of which run along the [205] direction, which is one of the approximate axes of thermal expansion.

The analysis of the distances between molecules in the TPH-ACID dimer, as illustrated in Figure 13b, is associated with NTE along the approximate axis of thermal expansion [201̅]. The thermal expansion parameters in the 300–415 and 350–415 K ranges are −423.6156 and −696.5488 MK^–1^, respectively, indicating a colossal negative thermal expansion. The distances between C3···C6, C3···C9, and O4···O4 atoms were considered. The observed increase in distance between the oxygen atoms O4 of the carboxyl groups of acid molecules with rising temperature may relate to changes in the COOH group’s conformation relative to the acid’s aromatic ring. Conversely, the distance between theophylline and pyromellitic acid molecules, characterized by the distances C3···C6 and C3···C9, decreases as the temperature increases. The observed reduction of the C3···C9 parameter from 6.049(3) Å at 300 K to 5.693(12) Å at 415 K, and the C3···C6 parameter from 4.986(3) Å at 300 K to 4.704(11) Å at 415 K, indicates that the TPH and PMLA molecules come closer together as the temperature rises, consistent with the observed negative thermal expansion along the [201̅] direction.

The hydrogen bonds forming the TPH-ACID heterosynthon occur along the [205] direction, which is approximately one of the axes of thermal expansion determined by using PASCAL software. The thermal expansion parameters along this direction in the 300–415 and 350–415 K ranges are positive, and equal to 43.90 and 61.88 MK^–1^, respectively. An analysis of the hydrogen bonds forming this heterosynthon, particularly the D···A length, was significantly hindered by the possibility that the carboxyl group occupying two positions in the unit cell (static disorder). Changes in these parameters with increasing temperature do not exhibit a clear trend, as illustrated in Figure 13c. Aside from the parameters derived from the structure obtained at 415 K, at which the single crystal was initially powdered, affecting data quality – the D···A parameter increased with the temperature rise from 300 to 410 K, except for N3···O3, where this value decreased.

An increase in the temperature causes the release of methanol molecules from the crystal lattice (Figure 14), which is confirmed by both STA and X-ray structural analysis. A change in the stoichiometry of the TPH·PMLA·MeOH cocrystal solvate from 2:1:2 to 2:1:1 is observed when the single crystal was heated to 390 K (Figure 15). Heating the single crystal to 415 K causes a significant decrease in indexation and initial powdering of the crystal. At this stage, after performing data reduction for reflections with *I*/σ­(*I*) > 8, the structure refinement process showed the presence of two methanol molecules per unit cell. The DSC experiment indicates the complete loss of methanol at 430 K (Figure 11d). Additionally, at the final stage of X-ray experiment, the crystal was heated to 420 K and a powder X-ray diffraction experiment was conducted (Figure 15). The reflection positions for the obtained powder pattern coincide with the reflection positions of the TPH·PMLA 2:1 **II** cocrystal, which means that the desolvation process leads to the phase transition from the TPH·PMLA·MeOH 2:1:2 cocrystal solvate into the TPH·PMLA 2:1 **II** cocrystal.

**Figure 14:**
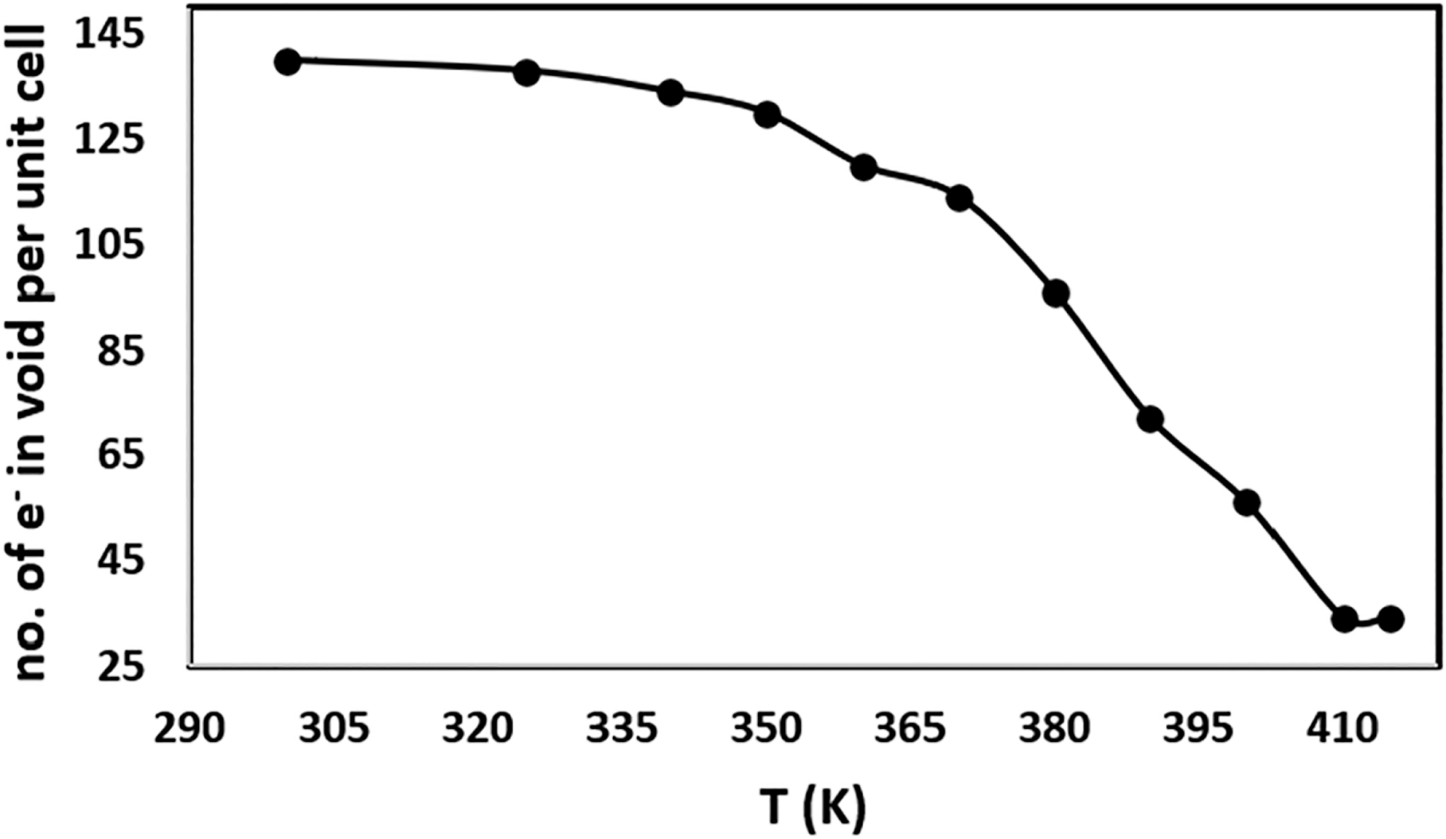
Graph of changes in the number of electrons per accessible void volume in the unit cell as a function of the temperature change.

**Figure 15:**
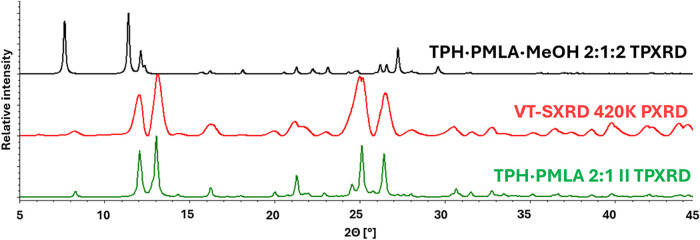
Comparison of theoretical powder patterns of the TPH·PMLA·MeOH 2:1:2 (black) and TPH·PMLA 2:1 **II** (green) forms with the diffraction pattern obtained for the pulverized crystal at 420 K (red).

### 3.6 Biological Studies

An analysis of the results illustrating the effect of selected bacterial species on TBR indicates interaction only with the *E. coli* strain, giving a zone of inhibition of bacterial growth of 6 mm. On the other hand, the remaining bacterial strains did not show any interaction with the tested compound. However, the synthesized TBR·PMLA 2:1 cocrystal showed a stronger effect on individual bacterial species, especially on *P. fluorescens*, limiting the growth of the strain to 6.5 mm (Table 2). In the case of this complex, *E. coli* turned out to be less sensitive, with a growth inhibition zone of only 3 mm. The TPH and PMLA compounds had a similar effect on the tested bacterial species, most strongly limiting the growth of *E. coli* (5 mm). However, the synthesized TPH·PMLA 2:1 cocrystal (both forms I and II), and TPH·PMLA·H_2_O 1:1:2 have a weaker effect on the tested bacteria than PMLA acid itself, which is particularly noticeable in the case of *M. luteus* and *B. subtilis* strains.

**Table 2:** Zone of Inhibition of Bacterial Growth (mm) for TBR, TPH, and PMLA Components, and TBR-PMLA and TPH-PMLA Systems

	zone of inhibition of bacterial growth (mm)			
compound	*M. luteus*	*B. subtilis*	*P. fluorescens*	*E. coli*
**TBR**	0	0	0	6
**TPH**	4	1	0	5
**PMLA**	3	3	0	5
**TBR·PMLA 2:1**	1	0.5	6.5	3
**TPH·PMLA 2:1 I**	0	1	0	5
**TPH·PMLA 2:1 II**	0	1.5	0	4
**TPH·PMLA·H**_**2**_ **O** **1:1:2**	0	1.5	0	4

When analyzing the effect of the tested compounds (TBR, TPH, and PMLA) on selected fungal species, the strongest inhibition of *F. culmorum* growth was observed, especially under the influence of PMLA (the zone of inhibition of mold development was 20 mm) and under the influence of TBR (inhibition zone = 17.5 mm). Comparing the analyzed compounds, it was found that TPH most strongly inhibited the development of most of the tested fungal species (Table 3). The formation of a TPH cocrystal with PMLA acid in some cases limits the growth of fungi more effectively than individual compounds (e.g., in the case of*F. culmorum*); however, it does not clearly indicate an increased inhibitory effect on the tested molds. A similar trend was also observed in the case of the TBR·PMLA 2:1 cocrystal, which in most cases did not have a significantly stronger toxic effect on individual mold species than TBR and PMLA alone.

**Table 3:** Zone of Inhibition of Fungal Growth (mm) for TBR, TPH, and PMLA Components, and TBR-PMLA and TPH-PMLA systems

	zone of inhibition of fungal growth (mm)					
compound	*F. culmorum*	*F. graminearum*	*T. atroviridae*	*T. harzianum*	*A. alternata*	*B. cinerea*
**TBR**	17.5	14	4	10	11	0
**TPH**	11	14	15	9	11.5	10
**PMLA**	20	11	10	9	10	9
**TBR·PMLA 2:1**	0	13	11.5	13.5	11.5	11
**TPH·PMLA 2:1 I**	14.5	13	16.5	12	10.5	13
**TPH·PMLA 2:1 II**	16	13	4	10	10	11
**TPH·PMLA·H**_**2**_ **O** **1:1:2**	16	12	15	4	10	10

The vast majority of biological studies focus on the antimicrobial potential of newly synthesized compounds against pathogenic or potentially pathogenic microorganisms. Promising results of inhibiting the growth of the opportunistic bacterial species *P. fluorescens* under the influence of the TBR·PMLA 2:1 cocrystal and *F. culmorum* under the influence of the TPH cocrystal with PMLA indicate the significant potential of newly synthesized compounds, whose properties may have real application possibilities, particularly in the medical sector.

The research conducted in this work additionally supplements existing knowledge regarding the effects of the tested compounds on environmental microbiota (*M. luteus*, *B. subtilis*, *T. atroviridae*, *T. harzianum*), which is undoubtedly important due to the possible presence of postapplication residues of these compounds in the natural environment. It should be noted that many compounds used, particularly in the pharmaceutical industry, penetrate natural ecosystems to varying degrees, often causing significant modifications to these environments.

## 4 Conclusions

As anticipated, benzene-1,2,4,5-tetracarboxylic acid (PMLA) forms a series of multicomponent crystal forms with theobromine (TBR) and theophylline (TPH). All five systems were obtained as single crystals, allowing for complete structural characterization. This analysis revealed the formation of various types of supramolecular synthons of the alkaloid–alkaloid, alkaloid–acid, or acid–acid types, which are responsible for the arrangement of molecules in the crystal lattice. Cocrystals of selected purine alkaloids were produced through mechanochemical syntheses carried out in a ball mill and using microwave radiation in a microwave reactor. The resulting phases were identified using powder X-ray diffraction. Grindings were performed in the absence or with a small volume of solvent for 30 min to 2 h. In this way, TBR·PMLA 2:1 cocrystals, both TPH·PMLA 2:1 **I** and **II** cocrystal polymorphs and the TPH·PMLA·H_2_O 1:1:2 cocrystal hydrate were successfully synthesized. Water proved to be the most effective solvent for microwave-assisted cocrystallization of TBR and PMLA, with almost complete conversion of the substrates at 125 °C in 10 min. The use of microwave radiation shortened the cocrystallization process using TPH and PMLA to 5 min, enabling the successful formation of both polymorphs of the TPH·PMLA 2:1 cocrystal and TPH·PMLA·H_2_O 1:1:2. On the other hand, the TPH·PMLA·MeOH 2:1:2 phase was identified only during cocrystallization from the solution.

The TBR·PMLA 2:1 cocrystal is almost 4.5 times more soluble in water than pure TBR and demonstrates thermal stability up to 203 °C. It exhibits a stronger bacterial inhibition than theobromine, particularly evident with *P. fluorescens*. This cocrystal also inhibits the growth of certain fungi, especially *B. cinerea*, more effectively than TBR. The cocrystallization of TPH with PMLA decreased the alkaloid’s solubility in water. Thermal studies indicated the stability of TPH·PMLA 2:1 cocrystals up to 205 °C. For solvates, STA studies confirmed the presence of a solvent within the crystal lattice, which was crucial for TPH·PMLA·MeOH 2:1:2 due to the challenges in determining the precise positions of the solvent molecules. Furthermore, the VT-SXRD experiment conducted in the 300–415 K range revealed the negative volumetric thermal expansion of TPH·PMLA·MeOH 2:1:2, possibly due to structural factors. Moreover, a phase transition to the TPH·PMLA 2:1 **II** form occurred, preceded by the gradual release of solvent molecules from TPH·PMLA·MeOH phase. Multicomponent systems containing TPH and PMLA were less effective in inhibiting bacterial growth than the individual components. In some instances, these systems more effectively limit fungal growth than the individual components; however, the effect of cocrystallization on inhibiting fungal growth remains inconclusive.

The subject of this highly relevant article is current and addresses the challenge of finding new multicomponent solid forms of active pharmaceutical ingredients (APIs). These studies are primarily of interest to the pharmaceutical industry due to the significant influence of cocrystallization on physicochemical properties without altering pharmacological properties. This work demonstrates the impact of benzene-1,2,4,5-tetracarboxylic acid on the cocrystallization of theobromine and theophylline. Theobromine is not widely utilized due to its poor solubility in water. Conversely, theophylline, which is used in treating respiratory diseases, exhibits good solubility in water but can convert into a hydrated form, negatively impacting the development of a consistent processing method for this API. The results obtained confirm the enhancement of theobromine solubility and the reduction of theophylline solubility in water after cocrystallization using pyromellitic acid as a coformer. Thermal studies have indicated the high thermal stability of unsolvated systems. Our findings also verified the biological activity of the tested systems against selected strains of bacteria and fungi. Furthermore, multicomponent systems containing purine alkaloids were synthesized through mechanochemical methods and microwave treatment within a short time frame and with minimal solvent usage, aligning with the principles of green chemistry. Such a strategy is advantageous for large-scale production, significantly faster than solution-based methods. Additionally, the rising drug resistance in humans to many pharmaceutical substances, alongside the growing global population, further justifies the search for safe drug formulations.

## Supplementary Material



## Notes

Solution-based, grinding, and microwave-assisted slurry cocrystallization conditions, powder patterns for obtained solids, crystallographic data from LT-SXRD and HT-SXRD data collections, steady-state absorption curves from UV–vis measurements, ORTEP drawings, hydrogen bonds geometry in the described cocrystals, and analysis of thermal expansion coefficients for TPH·PMLA·MeOH 2:1:2 system (PDF)
