# Supplementary material for: The Potential of Microwave Radiation and Mechanochemistry in the Formation of Purine Alkaloids Cocrystals Using Pyromellitic Acid as a Coformer: Synthesis, Structural, Spectroscopic, Thermal Analysis, and Biological Properties
Source: Cryst Growth Des. 2025 Aug 27;25(18):7684–700. doi: 10.1021/acs.cgd.5c00860 (PMC12447489; doi:10.1021/acs.cgd.5c00860)
Supplement: Supplementary file 1 [file cg5c00860_si_001.pdf]

The Potential of Microwave Radiation and Mechanochemistry in the Formation of Purine Alkaloids Cocrystals Using Pyromellitic Acid as a Coformer: Synthesis, Structural, Spectroscopic, Thermal Analysis, and Biological Properties

Weronika Cal[a)], Mateusz R. Gołdyn[a),b)], Oliwia Grupa [a)], Justyna Starzyk[c)],*

Daria Larowska-Zarych[d)], Kamil Frąckowiak[a)], Elżbieta Bartoszak-Adamska[a)]

[a] Faculty of Chemistry, Adam Mickiewicz University in Poznań, Uniwersytetu Poznańskiego 8, 61-614 Poznań, Poland

[b] Center for Advanced Technologies, Adam Mickiewicz University in Poznań, Uniwersytetu Poznańskiego 10, 61-614 Poznań, Poland

[c] Faculty of Agronomy, Horticulture, and Bioengineering, University of Life Science, Szydłowska 50, 60-656 Poznań, Poland

[d] Institute of Physical Chemistry, Polish Academy of Sciences, Kasprzaka 44/52, 01-224 Warsaw, Poland

* e-mail: mateusz.goldyn@amu.edu.pl

Supplementary

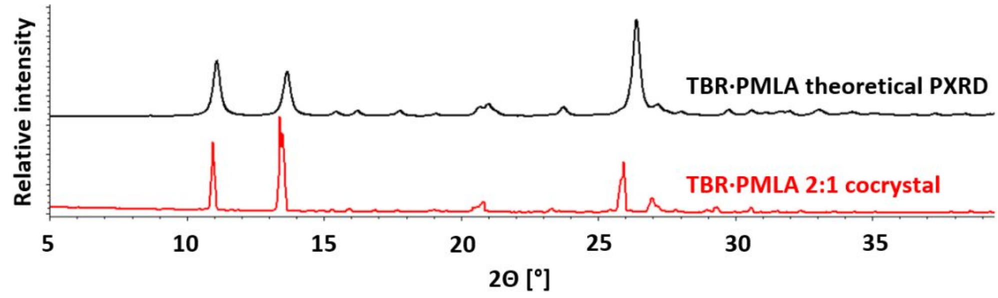

Fig. S1. The comparison of theoretical powder diffractogram with powder pattern of TBR·PMLA 2:1 phase obtained by slow evaporation from solution.

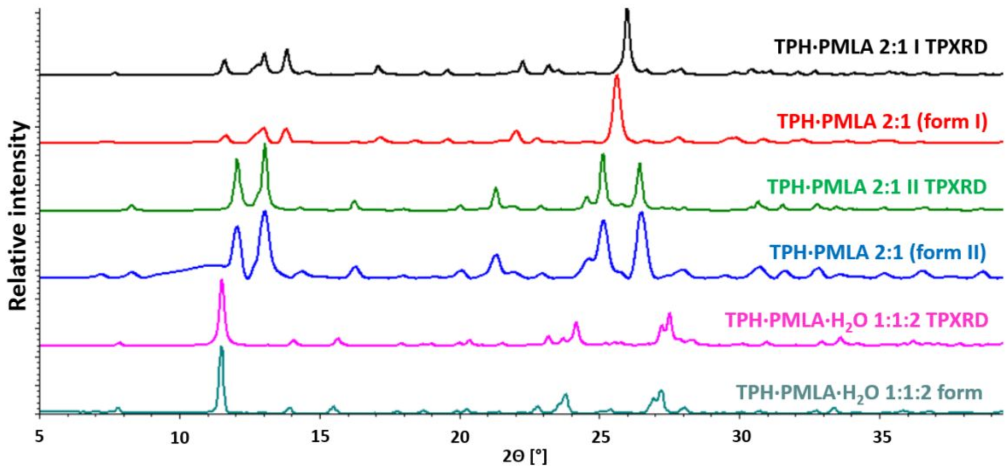

Fig. S2. The comparison of theoretical powder diffractograms with powder patterns for TPH-PMLA phases obtained by slow evaporation from solution.

Tab. S1. Details on conditions for solution cocrystallization.

	TBR/TPH	PMLA	Solvent, v/v, total volume
**TBR·PMLA 2:1 I**	18.6 mg (0.103 mmol)	13.4 mg (0.053 mmol)	MeCN – H_2O, 1:1, 10 mL
**TPH·PMLA 2:1 I**	24.8 mg (0.138 mmol)	17.5 mg (0.069 mmol)	MeOH – $MeNO_2$, 1:1, 8 mL
**TPH·PMLA 2:1 II**	25.0 mg (0.139 mmol)	17.7 mg (0.070 mmol)	MeOH – TCM, 1:1, 6 mL
**TPH·PMLA·MeOH 2:1:2**	34.6 mg (0.192 mmol)	24.4 mg (0.096 mmol)	MeOH – $MeNO_2$, 1:1, 2 mL
**TPH·PMLA·H_2O 1:1:2**	14.8 mg (0.082 mmol)	21.1 mg (0.083 mmol)	MeCN – H_2O, 1:1, 8 mL

Tab. S2. Details on conditions for mechanochemical cocrystallization of TBR with PMLA in a 2:1 stoichiometric ratio.

	TBR	PMLA	Grinding time	Volume of solvent
**NG**	17.9 mg (0.099 mmol)	12.6 mg (0.050 mmol)	0.5 h	–
**LAG H_2O**	18.6 mg (0.103 mmol)	13.1 mg (0.052 mmol)	1 h	20 µL
**LAG MeOH**	25.0 mg (0.139 mmol)	17.8 mg (0.070 mmol)	0.5 h	25 µL
**LAG MeCN**	25.2 mg (0.140 mmol)	17.8 mg (0.070 mmol)	0.5 h	25 µL
**LAG AcOEt**	25.0 mg (0.139 mmol)	17.7 mg (0.070 mmol)	0.5 h	25 µL
**LAG TCM**	25.2 mg (0.140 mmol)	17.7 mg (0.070 mmol)	1 h	25 µL

Tab. S3. Details on conditions for mechanochemical cocrystallization of TPH with PMLA in a 2:1 stoichiometric ratio.

	TPH	PMLA	Grinding time	Volume of solvent
**NG**	13.6 mg (0.075 mmol)	9.6 mg (0.038 mmol)	0.5 h	–
**LAG MeOH**	24.3 mg (0.135 mmol)	17.2 mg (0.068 mmol)	1 h	25 µL
**LAG MeCN**	25.1 mg (0.139 mmol)	17.7 mg (0.070 mmol)	0.5 h	25 µL
	24.9 mg (0.138 mmol)	17.8 mg (0.070 mmol)	1 h	25 µL
**LAG AcOEt**	25.5 mg (0.142 mmol)	18.2 mg (0.072 mmol)	0.5 h	25 µL
	17.9 mg (0.099 mmol)	12.6 mg (0.050 mmol)	1 h	25 µL
	23.0 mg (0.128 mmol)	16.3 mg (0.064 mmol)	2 h	25 µL
**LAG TCM**	24.9 mg (0.138 mmol)	17.5 mg (0.069 mmol)	0.5 h	25 µL

Tab. S4. Details on conditions for mechanochemical cocrystallization of TPH with PMLA in a 1:1 stoichiometric ratio.

	TPH	PMLA	Grinding time	Volume of solvent
**NG**	17.9 mg (0.099 mmol)	25.3 mg (0.100 mmol)	1 h	–
	36.6 mg (0.203 mmol)	51.7 mg (0.203 mmol)	2 h	–
**LAG H_2O**	18.1 mg (0.100 mmol)	25.5 mg (0.100 mmol)	1 h	20 µL
	38.3 mg (0.213 mmol)	54.0 mg (0.212 mmol)	2 h	35 µL

Tab. S5. Details on conditions for microwave-assisted slurry cocrystallization of TBR with PMLA in a 2:1 stoichiometric ratio.

	100°C / 5min	125°C / 10min	150°C / 10min	175°C / 10min
**H_2O**	tbr 19.7 mg (0.109 mmol)<br>pmla 14.0 mg (0.055 mmol)	tbr 38.9 mg (0.216 mmol)<br>pmla 27.3 mg (0.107 mmol)	tbr 31.1 mg (0.173 mmol)<br>pmla 21.9 mg (0.086 mmol)	tbr 19.9 mg (0.110 mmol)<br>pmla 14.1 mg (0.055 mmol)
**MeOH**	tbr 20.1 mg (0.112 mmol)<br>pmla 14.1 mg (0.055 mmol)	tbr 33.8 mg (0.188 mmol)<br>pmla 23.8 mg (0.094 mmol)	tbr 29.8 mg (0.165 mmol)<br>pmla 21.0 mg (0.083 mmol)	tbr 20.2 mg (0.112 mmol)<br>pmla 14.3 mg (0.056 mmol)
**MeCN**	tbr 19.9 mg (0.110 mmol)<br>pmla 13.9 mg (0.055 mmol)	tbr 35.7 mg (0.198 mmol)<br>pmla 25.3 mg(0.100 mmol)	tbr 30.3 mg (0.168 mmol)<br>pmla 21.5 mg (0.085 mmol)	–
**AcOEt**	tbr 20.0 mg (0.111 mmol)<br>pmla 14.1 mg(0.055 mmol)	tbr 35.7 mg (0.198 mmol)<br>pmla 25.3 mg (0.099 mmol)	tbr 30.7 mg (0.170 mmol)<br>pmla 21.7 mg (0.083 mmol)	–

Tab. S6. Details on conditions for microwave-assisted slurry cocrystallization of TPH with PMLA in a 2:1 stoichiometric ratio.

	75°C	100°C	125°C	150°C
**H_2O**	tph 27.3 mg (0.152 mmol)<br>pmla 19.1 mg (0.075 mmol)	tph 26.0 mg (0.144 mmol)<br>pmla 18.3 mg (0.072 mmol)	tph 25.4 mg (0.141 mmol)<br>pmla 17.9 mg (0.070 mmol)	tph 26.4 mg (0.147 mmol)<br>pmla 18.6 mg (0.073 mmol)
**MeOH**	tph 27.3 mg (0.152 mmol)<br>pmla 19.4 mg (0.076 mmol))	tph 28.5 mg (0.185 mmol)<br>pmla 20.1 mg (0.079 mmol)	tph 25.5 mg (0.142 mmol)<br>pmla 18.1 mg (0.071 mmol)	tph 26.3 mg (0.146 mmol)<br>pmla 18.5 mg (0.073 mmol)
**MeCN**	tph 27.7 mg (0.154 mmol)<br>pmla 19.6 mg (0.077 mmol)	tph 28.7 mg (0.159 mmol)<br>pmla 20.2 mg (0.079 mmol)	tph 25.9 mg (0.144 mmol)<br>pmla 18.4 mg (0.072 mmol)	tph 26.3 mg (0.146 mmol)<br>pmla 18.6 mg (0.073 mmol)
**AcOEt**	tph 26.6 mg (0.148 mmol)<br>pmla 18.7 mg (0.074 mmol)	tph 26.7 mg (0.148 mmol)<br>pmla 18.8 mg (0.074 mmol)	tph 24.6 mg (0.137 mmol)<br>pmla 17.4 mg (0.068 mmol)	tph 28.1 mg (0.156 mmol)<br>pmla 19.7 mg (0.078 mmol)

Tab. S7. Details on conditions for microwave-assisted slurry cocrystallization of TPH with PMLA in a 1:1 stoichiometric ratio.

	**75°C**	**100°C**	**125°C**
**H_2O**	tph 21.3 mg (0.118 mmol)<br>pmla 30.1 mg (0.118 mmol)	tph 21.8 mg (0.121 mmol)<br>pmla 30.8 mg (0.121 mmol)	tph 21.8 mg (0.121 mmol)<br>pmla 30.6 mg (0.120 mmol)
**MeOH**	tph 21.4 mg (0.119 mmol)<br>pmla 30.2 mg (0.119 mmol)	tph 21.4 mg (0.119 mmol)<br>pmla 30.2 mg (0.119 mmol)	tph 21.7 mg (0.120 mmol)<br>pmla 30.6 mg (0.120 mmol)
**MeCN**	tph 21.2 mg (0.118 mmol)<br>pmla 29.7 mg (0.117 mmol)	tph 21.3 mg (0.118 mmol)<br>pmla 30.1 mg (0.118 mmol)	tph 21.5 mg (0.119 mmol)<br>pmla 30.3 mg (0.119 mmol)
**AcOEt**	tph 21.6 mg (0.120 mmol)<br>pmla 30.5 mg (0.120 mmol)	tph 21.7 mg (0.120 mmol)<br>pmla 30.6 mg (0.120 mmol)	tph 21.6 mg (0.120 mmol)<br>pmla 30.5 mg (0.120 mmol)

Tab. S8. Relevant crystallographic data and refinement details for multicomponent systems containing PMLA.

**Cocrystal**	**TBR·PMLA 2:1**	**TPH·PMLA 2:1 I**	**TPH·PMLA 2:1 II**	**TPH·PMLA·H_2O 1:1:2**	**TPH·PMLA·MeOH 2:1:2**
**Empirical formula**	$C_{24}H_{22}N_8O_{12}$	$C_{24}H_{22}N_8O_{12}$	$C_{24}H_{22}N_8O_{12}$	$C_{17}H_{18}N_4O_{12}$	$C_{26}H_{30}N_8O_{14}$
**Formula weight/gmol^{-1}**	614.49	614.49	614.49	470.35	678.58
**Temperature/K**	131.3(2)	132.0(6)	133.6(3)	133.95(10)	133(3)
**Crystal system**	triclinic	triclinic	triclinic	orthorhombic	monoclinic
**Space group**	$P\bar{1}$	$P\bar{1}$	$P\bar{1}$	$Pca2_1$	$C2/c$
***a*/Å**	7.1801(5)	7.7054(3)	8.2042(7)	22.5347(4)	24.1806(8)
***b*/Å**	9.3186(8)	12.3787(6)	8.5305(7)	8.3906(1)	8.2335(3)
***c*/Å**	11.2905(6)	14.5102(7)	11.0866(6)	10.4885(2)	15.2968(6)
**α/°**	111.232(6)	109.976(4)	95.571(5)	90	90
**β/°**	95.322(5)	92.614(4)	102.892(6)	90	107.565(4)
**γ/°**	109.511(7)	96.851(4)	118.128(9)	90	90
***V*/Å^3**	643.78(9)	1285.92(11)	648.29(10)	1983.16(6)	2903.46(19)
Z, Z'	1, 0.5	2, 1	1, 0.5	4, 1	4, 0.5
**ρ_{calc}/gcm^{-3}**	1.585	1.587	1.574	1.575	1.552
**μ/mm^{-1}**	1.119	1.120	1.111	1.186	1.102
***F*(000)**	318.0	636.0	318.0	976.0	1416.0
**Crystal size/mm^3**	0.22 × 0.09 × 0.08	0.23 × 0.11 × 0.05	0.36 × 0.28 × 0.11	0.79 × 0.11 × 0.06	0.95 × 0.58 × 0.41
**Radiation/Å**	Cu *K*α (λ = 1.54184)	Cu *K*α (λ = 1.54184)	Cu *K*α (λ = 1.54184)	Cu *K*α (λ = 1.54184)	Cu *K*α (λ = 1.54184)
**2θ range/°**	8.662 to 152.37	6.51 to 152.638	8.418 to 151.976	7.846 to 151.808	7.67 to 152.402
**Index ranges**	$-8 \le h \le 7$<br>$-11 \le k \le 11$<br>$-14 \le l \le 12$	$-7 \le h \le 9$<br>$-14 \le k \le 15$<br>$-17 \le l \le 18$	$-9 \le h \le 10$<br>$-10 \le k \le 10$<br>$-12 \le l \le 13$	$-28 \le h \le 27$<br>$-10 \le k \le 7$<br>$-13 \le l \le 11$	$-30 \le h \le 25$<br>$-9 \le k \le 10$<br>$-19 \le l \le 18$
**Reflections collected**	4718	10128	4820	7723	6280
**Independent reflections**	2605 [R_{int} = 0.0270, R_{sigma} = 0.0373]	5234 [R_{int} = 0.0260, R_{sigma} = 0.0431]	2629 [R_{int} = 0.0242, R_{sigma} = 0.0279]	3225 [R_{int} = 0.0361, R_{sigma} = 0.0380]	2982 [R_{int} = 0.0250, R_{sigma} = 0.0236]
**Reflections with I ≥ 2σ (I)**	2160	4424	2353	2998	2724
**Data/restraints/parameters**	2605/0/244	5234/0/485	2629/0/243	3225/1/370	2982/48/259
**Final R indexes [I ≥ 2σ (I)]**	R_1 = 0.0376<br>wR_2 = 0.0936	R_1 = 0.0485<br>wR_2 = 0.1310	R_1 = 0.0396<br>wR_2 = 0.1088	R_1 = 0.0348<br>wR_2 = 0.0853	R_1 = 0.0650<br>wR_2 = 0.1791
**Final R indexes (all data)**	R_1 = 0.0474<br>wR_2 = 0.1020	R_1 = 0.0585<br>wR_2 = 0.1450	R_1 = 0.0440<br>wR_2 = 0.1131	R_1 = 0.0389<br>wR_2 = 0.0888	R_1 = 0.0693<br>wR_2 = 0.1821
**Goodness-of-fit on *F*^{2}**	1.040	1.041	1.049	1.084	1.123
**Largest diff. peak/hole/eÅ^{-3}**	0.27/-0.22	0.34/-0.27	0.30/-0.24	0.20/-0.21	0.60/-0.36
**CCDC deposit no.**	2427610	2427611	2427612	2427613	2427614

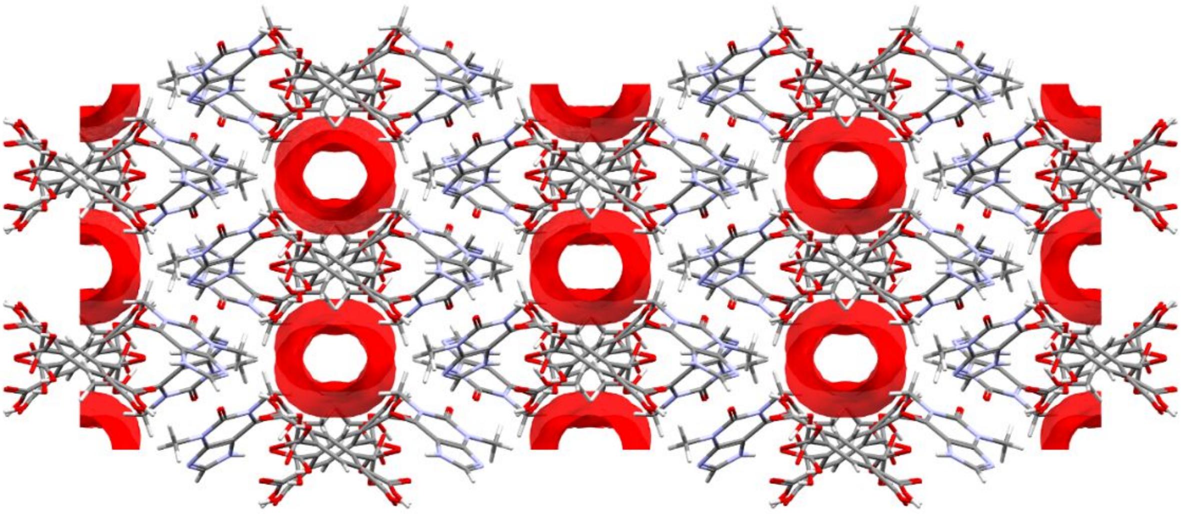

Fig. S3. Representation of VOIDs in TPH·PMLA·MeOH 2:1:2 crystal structure. VOID analysis was performed with Mercury software using the contact surface algorithm (probe radius of 1.2 Å and grid spacing of 0.3 Å).

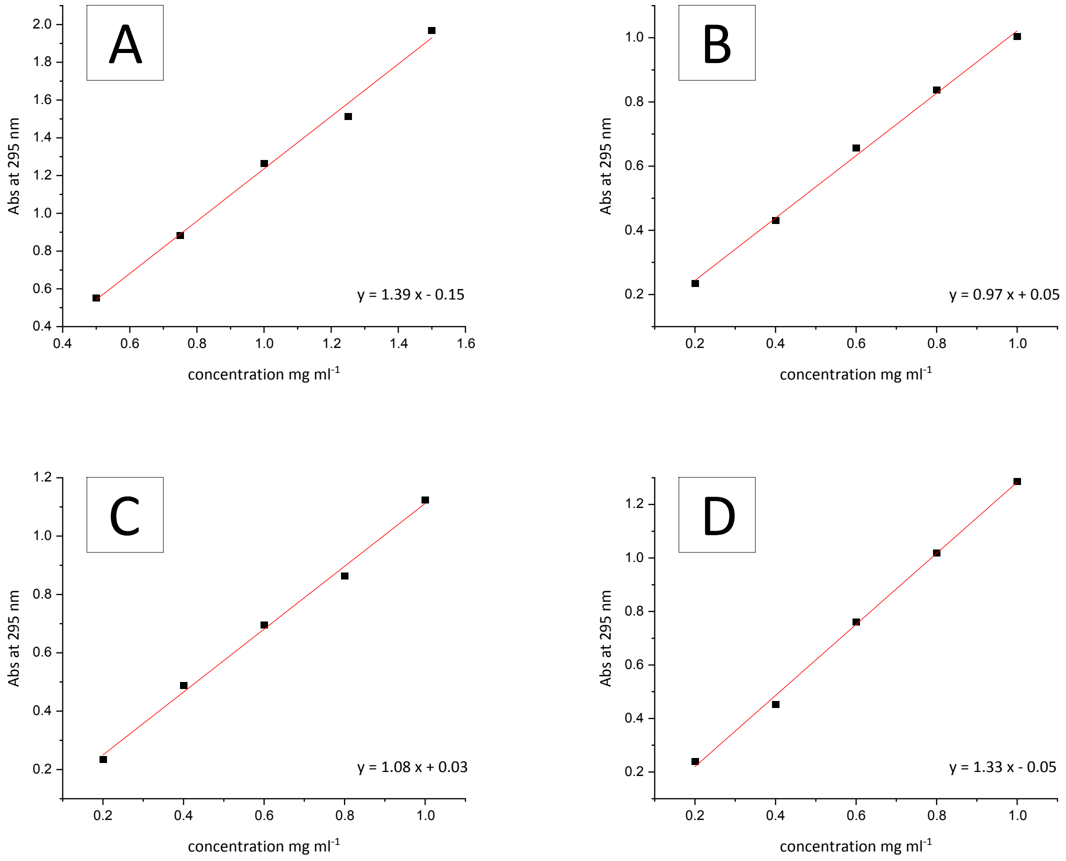

Fig. S4. Steady-state absorption calibration curves of (A) TPH·PMLA·H_2O 1:1:2, (B) TPH·PMLA 2:1 **I**, (C) TPH·PMLA 2:1 **II**, (D) TBR·PMLA 2:1.

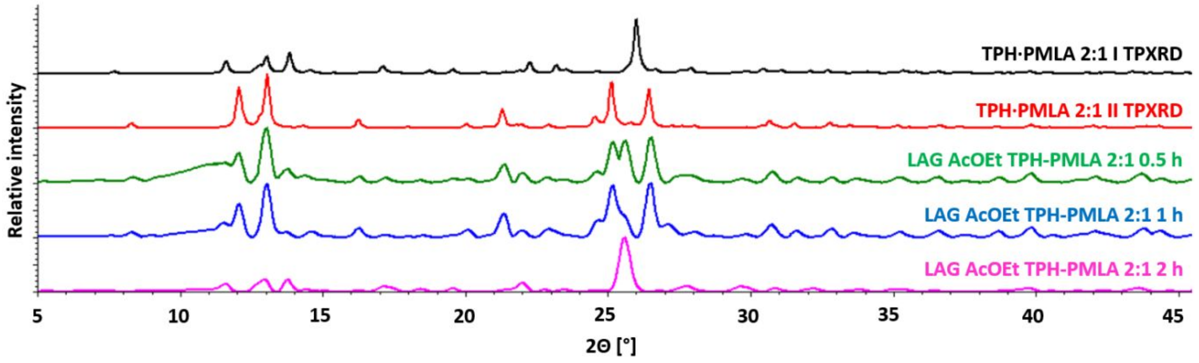

Fig. S5. The comparison of theoretical powder diffractograms with powder patterns for solids obtained by ethyl acetate-assisted grinding of TPH and PMLA in a 2:1 stoichiometric ratio conducted at different times (0.5h, 1h, 2h).

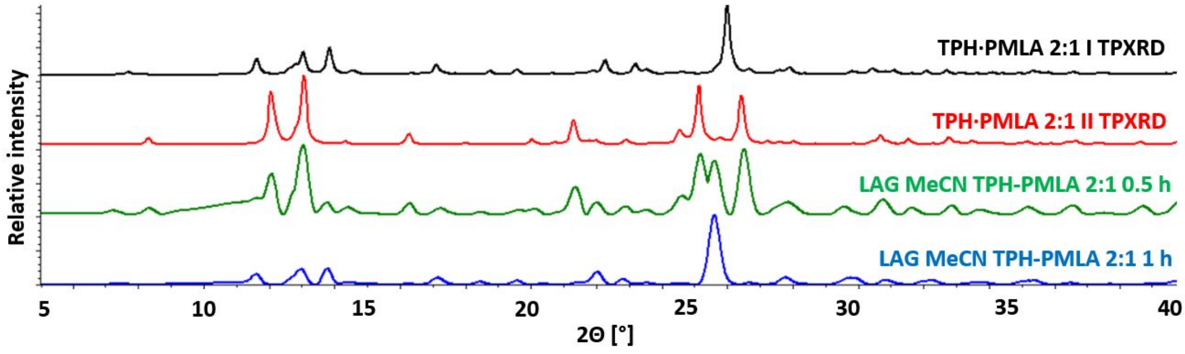

Fig. S6. The comparison of theoretical powder diffractograms with powder patterns for solids obtained by acetonitrile-assisted grinding of TPH and PMLA in a 2:1 stoichiometric ratio conducted at different times (0.5h, 1h).

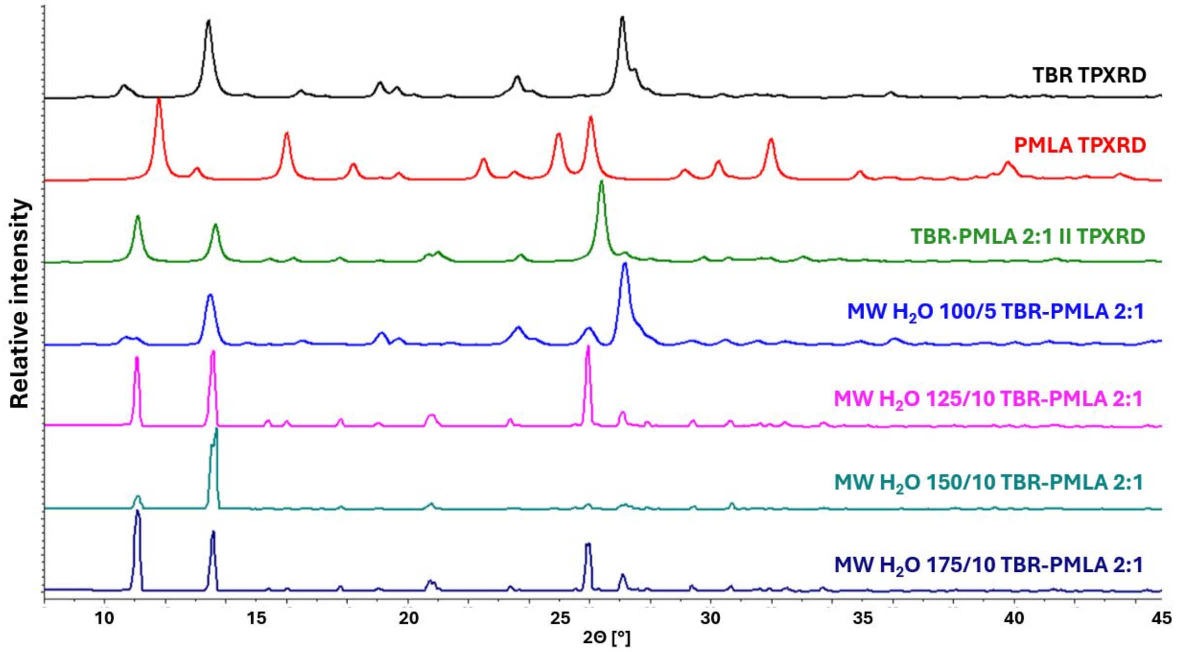

Fig. S7. The comparison of theoretical powder diffractograms of TBR, PMLA and TBR·PMLA 2:1 and with powder patterns for solids obtained by microwave-assisted slurry cocrystallization of TBR and PMLA in a 2:1 stoichiometric ratio performed in water.

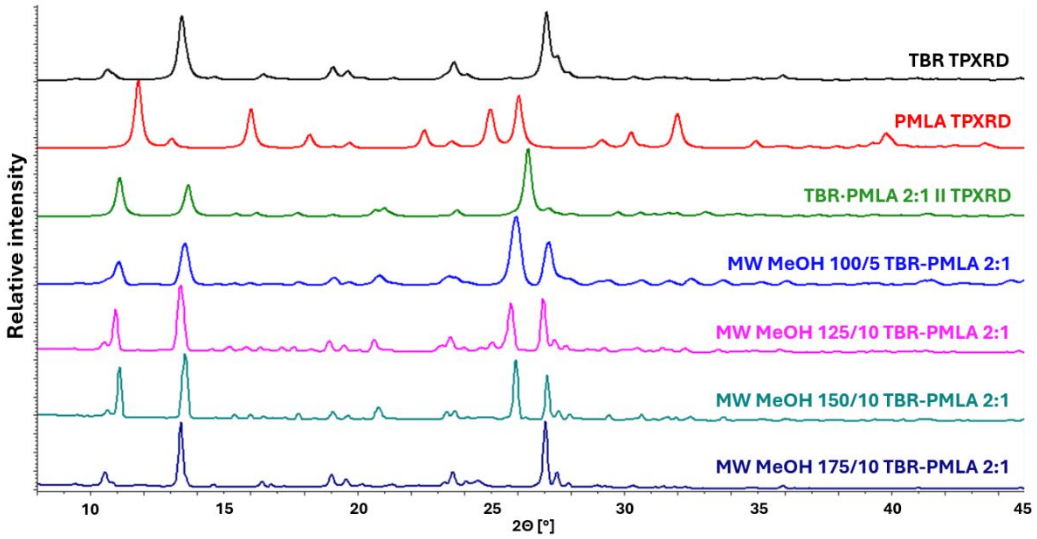

Fig. S8. The comparison of theoretical powder diffractograms of TBR, PMLA and TBR·PMLA 2:1 and with powder patterns for solids obtained by microwave-assisted slurry cocrystallization of TBR and PMLA in a 2:1 stoichiometric ratio performed in methanol.

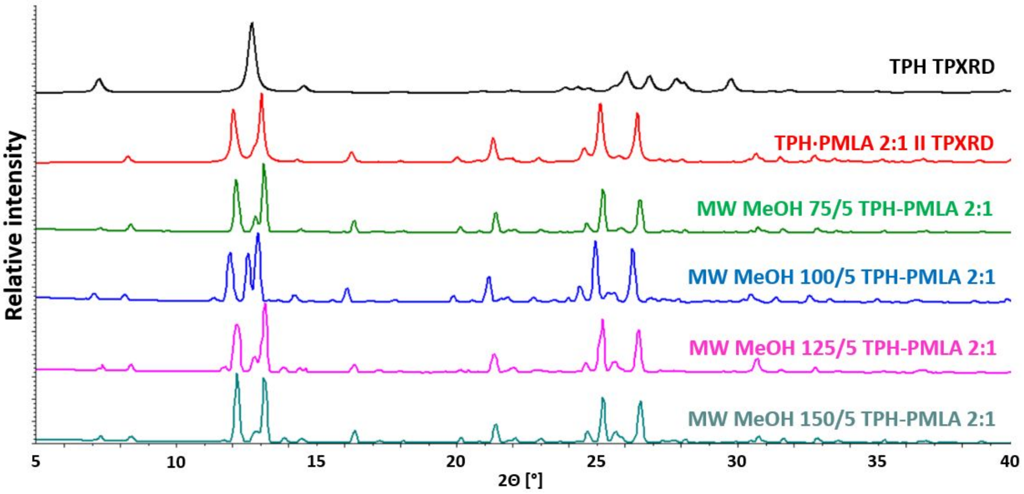

Fig. S9. The comparison of theoretical powder diffractograms of TPH and TPH·PMLA 2:1 form **II** with powder patterns for solids obtained by microwave-assisted cocrystallization of TPH and PMLA in a 2:1 stoichiometric ratio performed in methanol.

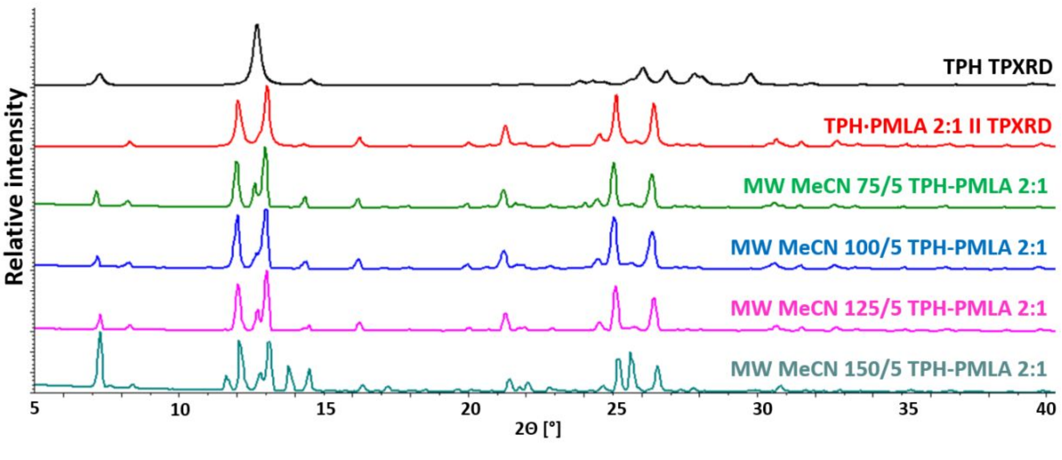

Fig. S10. The comparison of theoretical powder diffractograms of TPH and TPH·PMLA 2:1 form **II** with powder patterns for solids obtained by microwave-assisted cocrystallization of TPH and PMLA in a 2:1 stoichiometric ratio performed in acetonitrile.

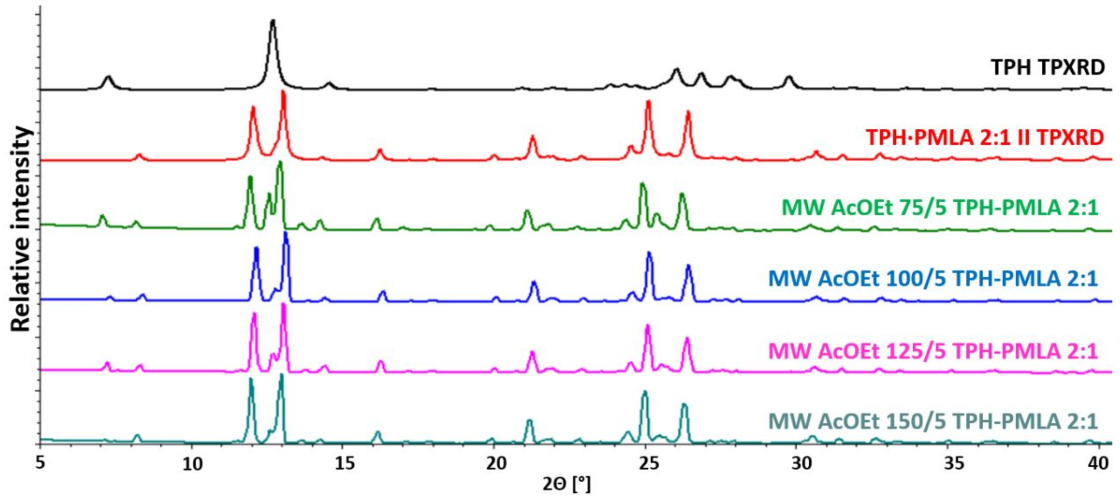

Fig. S11. The comparison of theoretical powder diffractograms of TPH and TPH·PMLA 2:1 form **II** with powder patterns for solids obtained by microwave-assisted cocrystallization of TPH and PMLA in a 2:1 stoichiometric ratio performed in ethyl acetate.

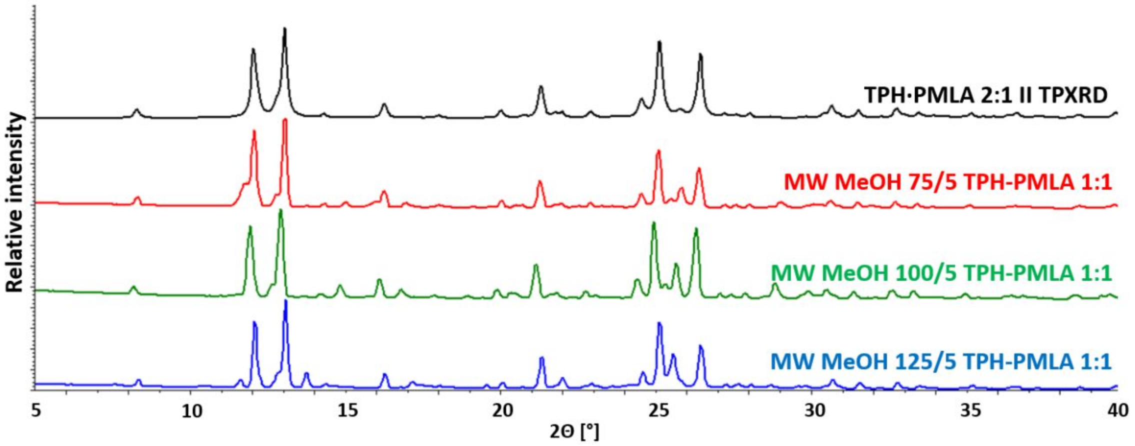

Fig. S12. The comparison of theoretical powder diffractograms of TPH·PMLA·H_2O 1:1:2 with powder patterns for solids obtained by microwave-assisted cocrystallization of TPH and PMLA in a 1:1 stoichiometric ratio performed in methanol.

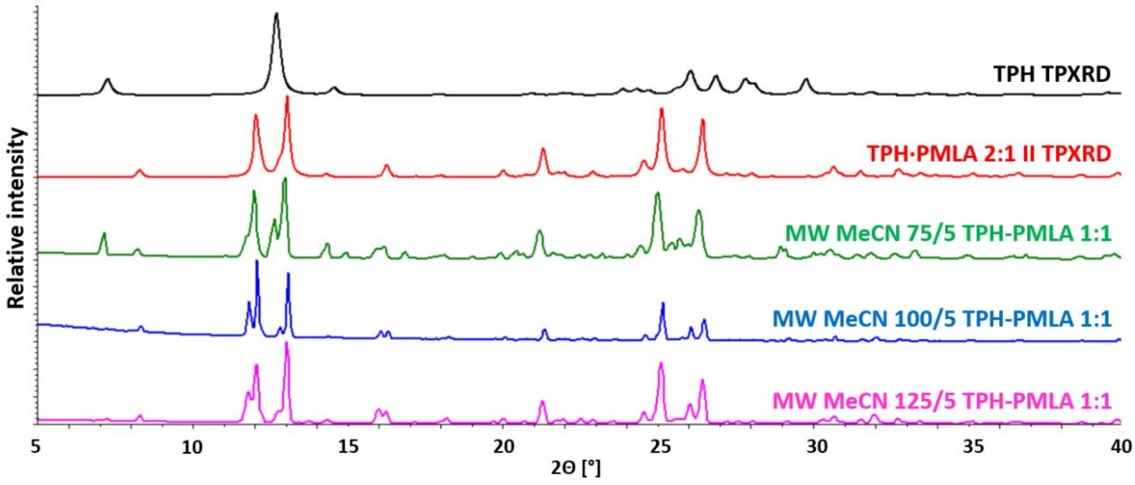

Fig. S13. The comparison of theoretical powder diffractograms of TPH·PMLA·H_2O 1:1:2 with powder patterns for solids obtained by microwave-assisted cocrystallization of TPH and PMLA in a 1:1 stoichiometric ratio performed in acetonitrile.

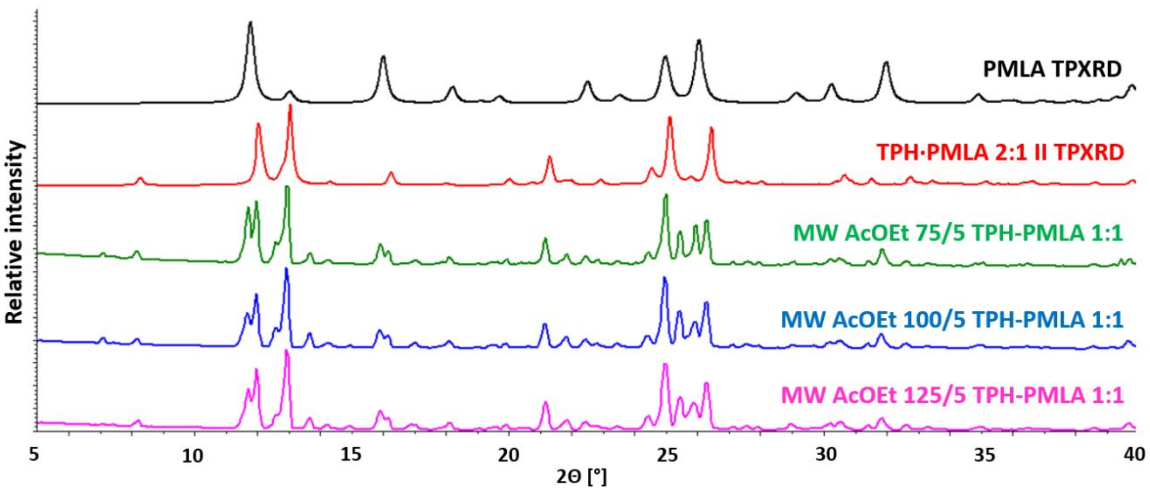

Fig. S14. The comparison of theoretical powder diffractograms of TPH·PMLA·H_2O 1:1:2 with powder patterns for solids obtained by microwave-assisted cocrystallization of TPH and PMLA in a 1:1 stoichiometric ratio performed in ethyl acetate.

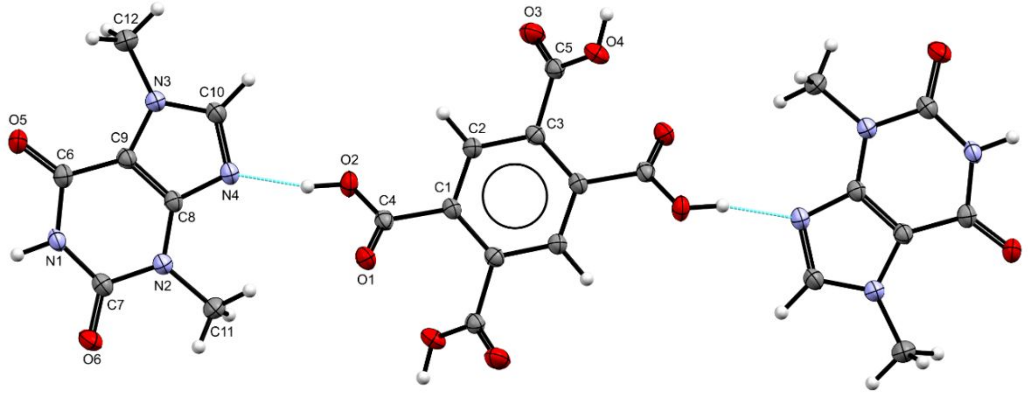

Fig. S15. ORTEP representation of the asymmetric unit of TBR·PMLA 2:1 cocrystal with the numbering of atoms belonging to the asymmetric unit. Ellipsoids were plotted with a 50% probability level. H-atoms are shown as spheres of arbitrary radii.

Tab. S9. Hydrogen bond parameters for TBR·PMLA 2:1 cocrystal.

D—H···*A*	*D*—H (Å)	H···*A* (Å)	*D*···*A* (Å)	*D*—H···*A* (°)
O2—H2···N4	0.93 (3)	1.73 (3)	2.6450 (17)	166 (3)
N1—H1···O5^{ii}	0.91 (3)	1.90 (3)	2.8074 (18)	170 (2)
O4—H4···O6^{i}	0.88 (3)	1.85 (3)	2.6497 (17)	150 (3)
Symmetry code(s): (i) *x*+2, *y*+1, *z*+1; (ii) −*x*−1, −*y*+1, −*z*.				

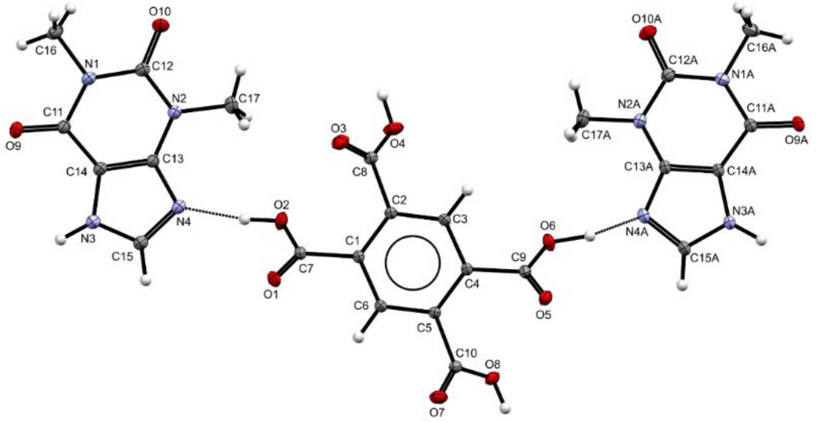

Fig. S16. ORTEP representation of the asymmetric unit of TPH·PMLA 2:1 **I** with the labeling of atoms. Ellipsoids were plotted with a 50% probability level. H-atoms are shown as spheres of arbitrary radii.

Tab. S10. Hydrogen bond parameters for TPH·PMLA 2:1 **I** cocrystal.

D—H···*A*	*D*—H (Å)	H···*A* (Å)	*D*···*A* (Å)	*D*—H···*A* (°)
O8—H8···O5^{i}	0.83 (3)	1.83 (3)	2.6557 (19)	170 (3)
N3A—H3A···O9A^{ii}	0.90 (3)	1.88 (3)	2.750 (2)	162 (2)
O4—H4···O10^{iii}	0.92 (4)	1.77 (4)	2.654 (2)	163 (3)
N3—H3B···O9^{iv}	0.91 (3)	1.81 (3)	2.724 (2)	177 (3)
O2—H2···N4	0.98 (4)	1.68 (4)	2.664 (2)	174 (4)
O6—H6···N4A	1.02 (4)	1.55 (4)	2.558 (2)	170 (4)
Symmetry code(s): (i) −*x*+2, −*y*, −*z*; (ii) −*x*+1, −*y*, −*z*−1; (iii) −*x*, −*y*+1, −*z*+1; (iv) −*x*+1, −*y*+1, −*z*+2.				

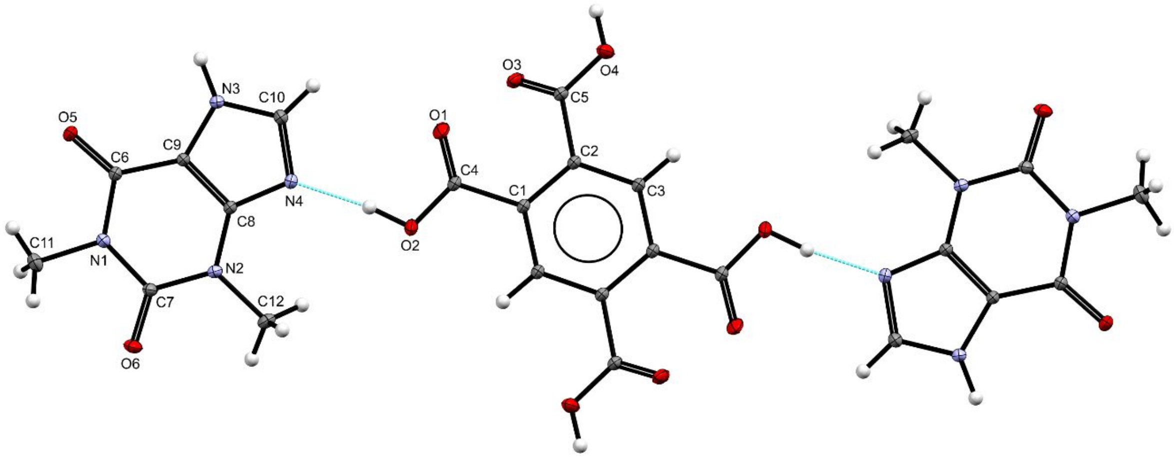

Fig. S17. ORTEP representation of the 2:1 fragment of TPH·PMLA 2:1 **II** with the labeling of atoms belonging to the asymmetric unit. Ellipsoids were drawn at a 50% probability level and hydrogen atoms are shown as spheres of arbitrary radii.

Tab. S11. Hydrogen bond parameters for TPH·PMLA 2:1 **II** cocrystal.

D—H···*A*	*D*—H (Å)	H···*A* (Å)	*D*···*A* (Å)	*D*—H···*A* (°)
O2—H2···N4	0.91 (3)	1.74 (3)	2.6478 (16)	176 (3)
N3—H3···O3^{i}	0.98 (3)	1.83 (3)	2.7906 (17)	165 (2)
O4—H4···O5^{ii}	0.94 (3)	1.73 (3)	2.6651 (16)	173 (3)
Symmetry code(s): (i) *x*, *y*−1, *z*−1; (ii) *x*, *y*+1, *z*+1.				

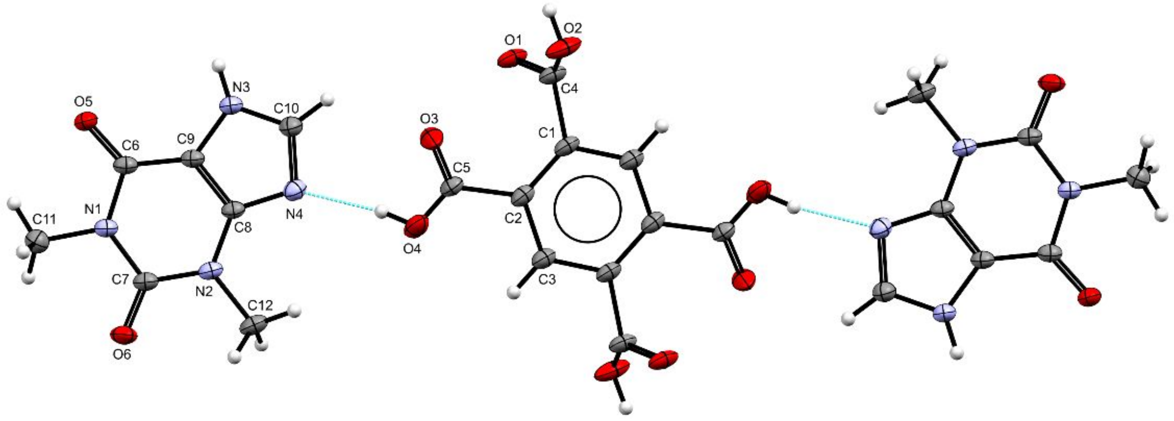

Fig. S18. ORTEP representation of the 2:1 fragment of TPH·PMLA·MeOH 2:1:2 with the numbering of atoms of the asymmetric unit. Ellipsoids were plotted with a 50% probability level. Hydrogen atoms were shown as spheres of arbitrary radii. Disordered carboxyl groups in pyromellitic acid molecule were omitted for clarity.

Tab. S12. Hydrogen bond parameters for TPH·PMLA·MeOH 2:1:2**.**

D—H···*A*	*D*—H (Å)	H···*A* (Å)	*D*···*A* (Å)	*D*—H···*A* (°)
O2—H2···N4	0.84	1.90	2.718 (17)	165.8
O2A—H2A···N4	0.84	1.82	2.662 (15)	178.2
O4—H4···O5^{ii}	0.84	1.79	2.618 (5)	166.4
O4A—H4A···O5^{ii}	0.84	1.85	2.641 (5)	157.4
N3—H3···O3^{i}	0.88	1.89	2.767 (4)	171.9
Symmetry code(s): (i) −*x*+1/2, *y*−1/2, −*z*+3/2; (ii) −*x*+1/2, *y*+1/2, −*z*+3/2. The oxygen (O2, O2A, O4, O4A) and hydrogen atoms (H2, H2A, H4, H4A) with fixed occupancy factors at 0.50.				

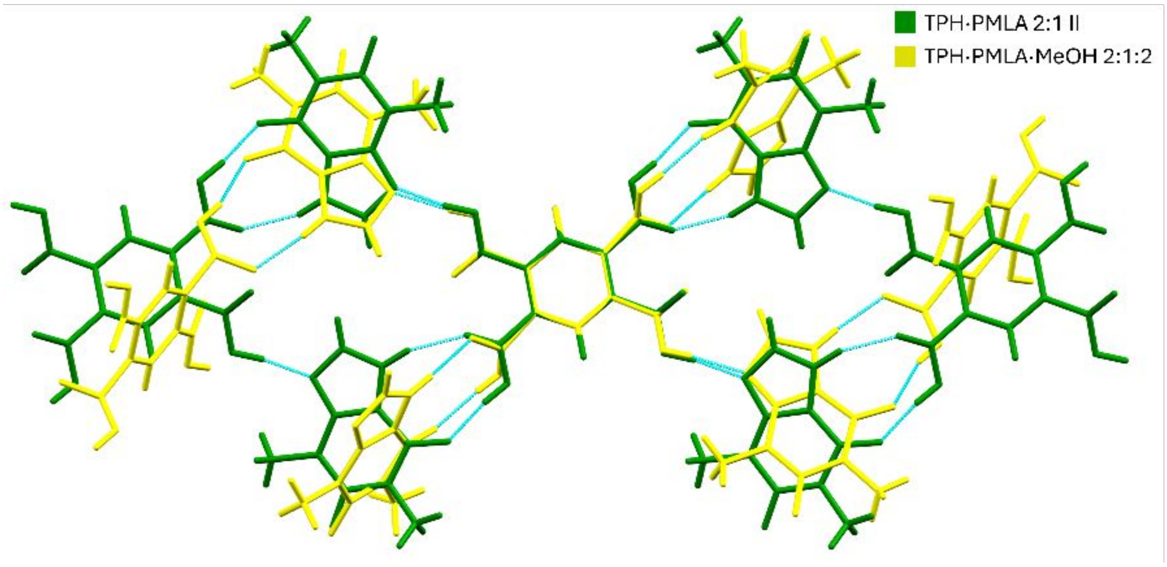

Fig. S19. Comparison of a fragment of the crystal lattice of the TPH·PMLA 2:1 **II** cocrystal and the TPH·PMLA·MeOH 2:1:2 cocrystal solvate by superimposing aromatic rings of PMLA molecules.

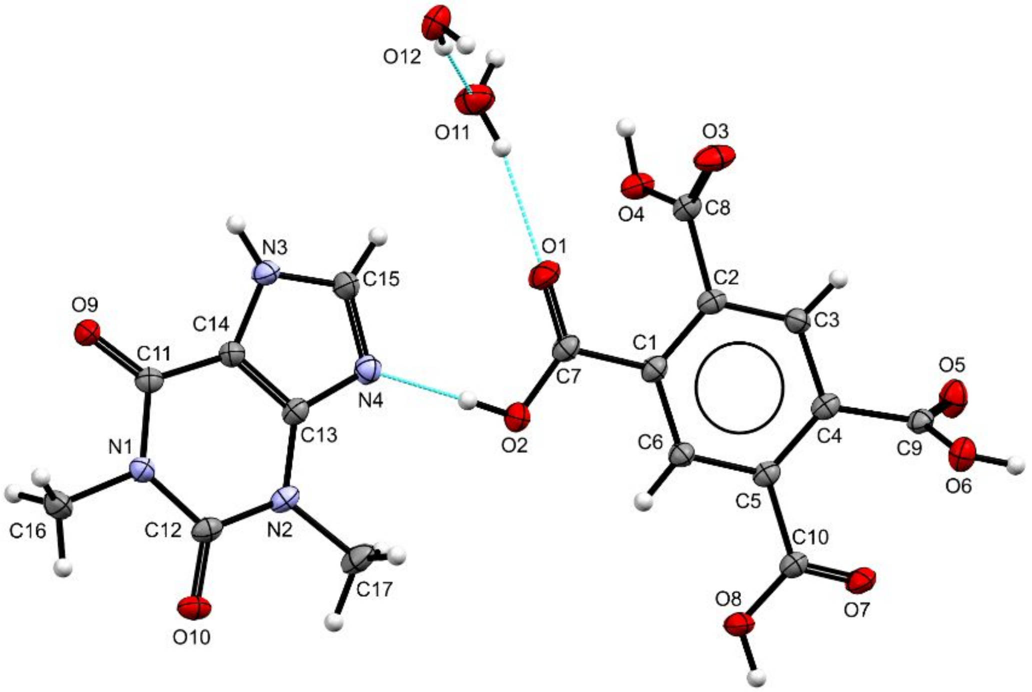

Fig. S20. ORTEP representation of the asymmetric unit of TPH·PMLA·H_2O 1:1:2 with the labeling of atoms. Ellipsoids were plotted with a 50% probability level. H-atoms are shown as spheres of arbitrary radii.

Tab. S13. Hydrogen bond parameters for TPH·PMLA·H_2O 1:1:2.

D—H···*A*	*D*—H (Å)	H···*A* (Å)	*D*···*A* (Å)	*D*—H···*A* (°)
O2—H2···N4	0.87 (5)	1.79 (5)	2.659 (4)	178 (5)
O4—H4···O10^{ii}	0.92 (7)	1.75 (7)	2.646 (3)	165 (6)
O6—H6···O12^{iii}	0.89 (7)	1.74 (7)	2.630 (4)	175 (6)
O8—H8···O9^{iii}	0.87 (6)	1.76 (6)	2.610 (3)	168 (5)
N3—H3A···O7^{i}	0.91 (5)	1.83 (5)	2.734 (4)	176 (5)
O11—H11A···O1	0.84 (6)	2.11 (6)	2.939 (4)	169 (5)
O11—H11B···O10^{ii}	0.89 (6)	2.10 (6)	2.902 (3)	149 (5)
O12—H12A···O11	0.81 (5)	1.97 (5)	2.777 (4)	173 (5)
O12—H12B···O9^{iv}	0.80 (7)	2.45 (6)	3.031 (4)	131 (5)
O12—H12B···O4^{v}	0.80 (7)	2.39 (6)	3.047 (4)	139 (5)
Symmetry code(s): (i) *x*, *y*−1, *z*+1; (ii) *x*+1/2, −*y*+1, *z*; (iii) *x*, *y*+1, *z*−1; (iv) −*x*+1/2, *y*, *z*−1/2; (v) *x*, *y*−1, *z*.				

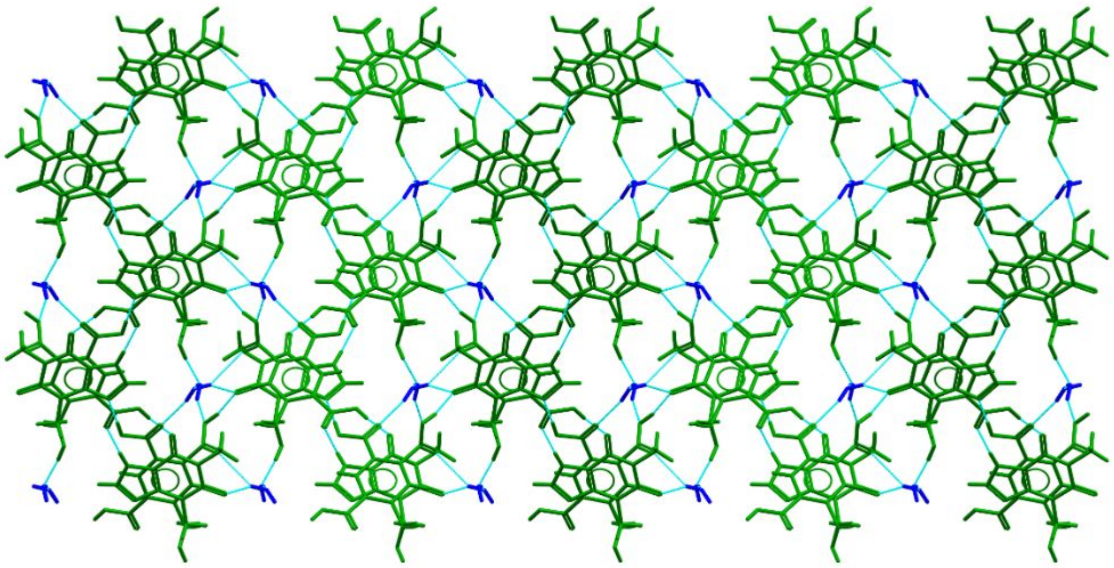

Fig. S21. 3D crystal structure of TPH·PMLA·H_2O 1:1:2 in a view along the [010] direction.

Tab. S14. Crystallographic data and refinement details for single crystal of TPH·PMLA·MeOH 2:1:X measured at 300, 325, 340 and 350 K.

**Temperature/K**	**300**	**325**	**340**	**350**
**Empirical formula**	$2C_7H_8N_4O_2{\cdot}C_{10}H_6O_8{\cdot}2CH_3OH$	$2C_7H_8N_4O_2{\cdot}C_{10}H_6O_8{\cdot}2CH_3OH$	$2C_7H_8N_4O_2{\cdot}C_{10}H_6O_8{\cdot}2CH_3OH$	$2C_7H_8N_4O_2{\cdot}C_{10}H_6O_8{\cdot}1.75CH_3OH$
**Formula weight/gmol^{-1}**	678.58	678.58	678.58	670.57
**Crystal system**	monoclinic	monoclinic	monoclinic	monoclinic
**Space group**	*C*2/*c*	*C*2/*c*	*C*2/*c*	*C*2/*c*
***a*/Å**	24.1351(9)	24.1541(8)	24.0908(7)	24.0528(10)
***b*/Å**	8.4645(3)	8.4984(3)	8.5235(3)	8.5411(4)
***c*/Å**	15.3228(6)	15.3308(4)	15.3268(4)	15.3153(5)
**α/°**	90	90	90	90
**β/°**	107.679(4)	107.687(3)	107.568(3)	107.480(4)
**γ/°**	90	90	90	90
***V*/Å^3**	2982.5(2)	2998.21(17)	3000.39(16)	3001.0(2)
Z*, *Z'	4; 0.5	4; 0.5	4; 0.5	4; 0.5
**ρ_{calc}/gcm^{-3}**	1.511	1.503	1.502	1.484
**μ/mm^{-1}**	1.073	1.067	1.066	1.053
***F*(000)**	1416.0	1416.0	1416.0	1398.0
**Crystal size/mm^3**	0.43 × 0.38 × 0.32	0.43 × 0.38 × 0.32	0.43 × 0.38 × 0.32	0.43 × 0.38 × 0.32
**Radiation/Å**	Cu *K*α (λ = 1.54184)	Cu *K*α (λ = 1.54184)	Cu *K*α (λ = 1.54184)	Cu *K*α (λ = 1.54184)
**2θ range/°**	7.69 to 148.356	7.684 to 141.416	7.698 to 141.086	7.708 to 141.7
**Index ranges**	$-28 \le h \le 29$, $-10 \le k \le 10$, $-17 \le l \le 19$	$-28 \le h \le 29$, $-10 \le k \le 10$, $-17 \le l \le 18$	$-29 \le h \le 29$, $-10 \le k \le 10$, $-16 \le l \le 18$	$-29 \le h \le 29$, $-10 \le k \le 7$, $-16 \le l \le 18$
**Reflections collected**	5946	5215	5246	5461
**Independent reflections**	2972 [R_{int} = 0.0204, R_{sigma} = 0.0281]	2834 [R_{int} = 0.0215, R_{sigma} = 0.0298]	2828 [R_{int} = 0.0186, R_{sigma} = 0.0270]	2847 [R_{int} = 0.0195, R_{sigma} = 0.0280]
**Reflections with I ≥ 2σ (I)**	2479	2318	2372	2324
**Data/restraints/parameters**	2972/0/259	2834/0/259	2828/0/259	2847/48/259
**Final R indexes [I ≥ 2σ (I)]**	R_1 = 0.0547, wR_2 = 0.1618	R_1 = 0.0568, wR_2 = 0.1635	R_1 = 0.0541, wR_2 = 0.1537	R_1 = 0.0548, wR_2 = 0.1565
**Final R indexes (all data)**	R_1 = 0.0614, wR_2 = 0.1712	R_1 = 0.0642, wR_2 = 0.1719	R1 = 0.0607, wR2 = 0.1616	R_1 = 0.0624, wR_2 = 0.1664
**Goodness-of-fit on *F*^{2}**	1.039	1.061	1.030	1.056
**Largest diff. peak/hole/eÅ^{-3}**	0.29/-0.25	0.24/-0.24	0.23/-0.22	0.20/-0.21
**CCDC deposit no.**	2435404	2435405	2435406	2435407

Tab. S15. Crystallographic data and refinement details for single crystal of TPH·PMLA·MeOH 2:1:X measured at 360, 370, 380 and 390 K.

**Temperature/K**	**360**	**370**	**380**	**390**
**Empirical formula**	$2C_7H_8N_4O_2{\cdot}C_{10}H_6O_8$ $\cdot 1.75CH_3OH$	$2C_7H_8N_4O_2{\cdot}C_{10}H_6O_8$ $\cdot 1.5CH_3OH$	$2C_7H_8N_4O_2{\cdot}C_{10}H_6O_8$ $\cdot 1.25CH_3OH$	$2C_7H_8N_4O_2{\cdot}C_{10}H_6O_8$ $\cdot CH_3OH$
**Formula weight/gmol^{-1}**	670.57	662.56	654.55	646.54
**Crystal system**	monoclinic	monoclinic	monoclinic	monoclinic
**Space group**	*C*2/*c*	*C*2/*c*	*C*2/*c*	*C*2/*c*
***a*/Å**	23.9816(12)	23.9082(9)	23.7727(14)	23.6021(16)
***b*/Å**	8.5574(3)	8.5746(3)	8.6100(5)	8.6418(6)
***c*/Å**	15.3156(8)	15.2897(5)	15.2557(7)	15.2258(7)
**α/°**	90	90	90	90
**β/°**	107.327(5)	107.194(4)	106.873(6)	106.422(6)
**γ/°**	90	90	90	90
***V*/Å^3**	3000.4(3)	2994.36(19)	2988.2(3)	2978.8(3)
Z*, *Z'	4; 0.5	4; 0.5	4; 0.5	4; 0.5
**ρ_{calc}/gcm^{-3}**	1.484	1.470	1.455	1.442
**μ/mm^{-1}**	1.053	1.042	1.031	1.021
***F*(000)**	1398.0	1380.0	1362.0	1344.0
**Crystal size/mm^3**	0.43 × 0.38 × 0.32	0.43 × 0.38 × 0.32	0.43 × 0.38 × 0.32	0.43 × 0.38 × 0.32
**Radiation/Å**	Cu *K*α (λ = 1.54184)	Cu *K*α (λ = 1.54184)	Cu *K*α (λ = 1.54184)	Cu *K*α (λ = 1.54184)
**2θ range/°**	7.724 to 141.614	7.742 to 141.388	7.772 to 141.572	7.81 to 141.47
**Index ranges**	$-28 \le h \le 27$, $-10 \le k \le 10$, $-17 \le l \le 14$	$-28 \le h \le 29$, $-10 \le k \le 7$, $-16 \le l \le 18$	$-28 \le h \le 28$, $-10 \le k \le 10$, $-18 \le l \le 17$	$-28 \le h \le 27$, $-9 \le k \le 10$, $-18 \le l \le 15$
**Reflections collected**	5190	5289	5310	5651
**Independent reflections**	2374 [R_{int} = 0.0201, R_{sigma} = 0.0256]	2830 [R_{int} = 0.0226, R_{sigma} = 0.0294]	2829 [R_{int} = 0.0228, R_{sigma} = 0.0322]	2549 [R_{int} = 0.0252, R_{sigma} = 0.0275]
**Reflections with I ≥ 2σ (I)**	1940	2302	2162	2031
**Data/restraints/parameters**	2374/0/253	2830/0/259	2829/8/259	2549/48/259
**Final R indexes [I ≥ 2σ (I)]**	R_1 = 0.0506, wR_2 = 0.1558	R_1 = 0.0523, wR_2 = 0.1556	R_1 = 0.0531, wR_2 = 0.1563	R_1 = 0.0553, wR_2 = 0.1547
**Final R indexes (all data)**	R_1 = 0.0571, wR_2 = 0.1647	R_1 = 0.0597, wR_2 = 0.1649	R_1 = 0.0642, wR_2 = 0.1669	R_1 = 0.0642, wR_2 = 0.1644
**Goodness-of-fit on *F*^{2}**	1.087	1.052	1.054	1.059
**Largest diff. peak/hole/eÅ^{-3}**	0.18/-0.22	0.18/-0.23	0.17/-0.20	0.17/-0.21
**CCDC deposit no.**	2435408	2435409	2435410	2435411

Tab. S16. Crystallographic data and refinement details for single crystal of TPH·PMLA·MeOH 2:1:X measured at 400, 410 and 415 K.

**Temperature/K**	**400**	**410**	**415**
**Empirical formula**	$2C_7H_8N_4O_2{\cdot}C_{10}H_6O_8{\cdot}0.75CH_3OH$	$2C_7H_8N_4O_2{\cdot}C_{10}H_6O_8{\cdot}0.5CH_3OH$	$2C_7H_8N_4O_2{\cdot}C_{10}H_6O_8{\cdot}0.5CH_3OH$
**Formula weight/gmol^{-1}**	638.53	630.52	630.52
**Crystal system**	monoclinic	monoclinic	monoclinic
**Space group**	*C*2/*c*	*C*2/*c*	*C*2/*c*
***a*/Å**	23.3671(14)	23.1938(12)	23.1535(14)
***b*/Å**	8.6836(5)	8.7032(5)	8.6933(6)
***c*/Å**	15.1837(6)	15.1537(6)	15.1569(7)
**α/°**	90	90	90
**β/°**	105.912(5)	105.670(5)	105.494(5)
**γ/°**	90	90	90
***V*/Å^3**	2962.9(3)	2945.2(3)	2939.9(3)
Z*, *Z'	4; 0.5	4; 0.5	4; 0.5
**ρ_{calc}/gcm^{-3}**	1.431	1.422	1.425
**μ/mm^{-1}**	1.013	1.005	1.007
***F*(000)**	1326.0	1308.0	1308.0
**Crystal size/mm^3**	0.43 × 0.38 × 0.32	0.43 × 0.38 × 0.32	0.43 × 0.38 × 0.32
**Radiation/Å**	Cu *K*α (λ = 1.54184)	Cu *K*α (λ = 1.54184)	Cu *K*α (λ = 1.54184)
**2θ range/°**	7.868 to 141.384	7.918 to 141.314	7.924 to 123.274
**Index ranges**	$-28 \le h \le 23$, $-10 \le k \le 10$, $-18 \le l \le 15$	$-28 \le h \le 27$, $-10 \le k \le 9$, $-18 \le l \le 15$	$-22 \le h \le 22$, $-7 \le k \le 6$, $-10 \le l \le 16$
**Reflections collected**	5365	5132	1586
**Independent reflections**	2787 [R_{int} = 0.0190, R_{sigma} = 0.0268]	2759 [R_{int} = 0.0224, R_{sigma} = 0.0323]	590 [R_{int} = 0.0174, R_{sigma} = 0.0188]
**Reflections with I ≥ 2σ (I)**	2150	2061	590
**Data/restraints/parameters**	2787/48/259	2759/48/259	590/52/259
**Final R indexes [I ≥ 2σ (I)]**	R_1 = 0.0581, wR_2 = 0.1672	R_1 = 0.0550, wR_2 = 0.1621	R_1 = 0.0461, wR_2 = 0.1058
**Final R indexes (all data)**	R_1 = 0.0692, wR_2 = 0.1811	R_1 = 0.0679, wR_2 = 0.1761	R_1 = 0.0461, wR_2 = 0.1058
**Goodness-of-fit on *F*^{2}**	1.065	1.067	1.135
**Largest diff. peak/hole/eÅ^{-3}**	0.18/-0.21	0.17/-0.20	0.14/-0.24
**CCDC deposit no.**	2435412	2435413	2435414

Tab. S17. The values of volumetric thermal expansion coefficients α_V (MK^{-1}) with error parameter $\sigma\alpha_V$ (MK^{-1}) for TPH·PMLA·MeOH 2:1:2 calculated for different temperature ranges.

Temperature range (K)	α_V (MK^{-1})	$\sigma\alpha_V$ (MK^{-1})
300 – 350	131.7605	16.6462
350 – 415	-336.0357	36.8181
300 – 415	-135.0891	59.3959

Tab. S18. Principal coefficients of thermal expansion and corresponding principal axes determined for TPH·PMLA·MeOH 2:1:2 in different temperature ranges.

Temperature range (K)	Principal axis, *i*	α_i (MK^{-1})	$\sigma\alpha_i$ (MK^{-1})	Component of x_i along the crystallographic axes			Approximate axis
				a	*b*	*c*	
300 – 350	1	21.993	2.021	0.2391	-0.0	0.971	[104]
	2	-68.5182	19.5007	0.9889	-0.0	-0.1487	$[70\bar{1}]$
	3	178.0334	4.0565	-0.0	1.0	-0.0	[010]
350 – 415	1	-696.5488	43.1709	0.8577	-0.0	-0.5141	$[20\bar{1}]$
	2	61.8835	8.255	0.363	0.0	0.9318	[205]
	3	310.831	20.269	-0.0	1.0	0.0	[010]
300 – 415	1	43.9009	5.7608	0.3424	-0.0	0.9396	[205]
	2	-423.6156	76.2655	0.8888	-0.0	-0.4583	$[20\bar{1}]$
	3	253.4889	16.7037	-0.0	1.0	0.0	[010]
